# Phenotyping for Nitrogen Use Efficiency: Rice Genotypes Differ in N-Responsive Germination, Oxygen Consumption, Seed Urease Activities, Root Growth, Crop Duration, and Yield at Low N

**DOI:** 10.3389/fpls.2018.01452

**Published:** 2018-10-01

**Authors:** Narendra Sharma, Vimlendu Bhushan Sinha, Neha Gupta, Soumya Rajpal, Surekha Kuchi, Vetury Sitaramam, Rajender Parsad, Nandula Raghuram

**Affiliations:** ^1^School of Biotechnology, Guru Gobind Singh Indraprastha University, Dwarka, India; ^2^Indian Institute of Rice Research, Hyderabad, India; ^3^Anant Cooperative Housing Society, Pune, India; ^4^Indian Agricultural Statistics Research Institute, Pusa, India

**Keywords:** NUE, phenotype, nitrogen, germination, yield, crop duration, root, oxygen

## Abstract

The biological improvement of fertilizer nitrogen use efficiency (NUE) is hampered by the poor characterization of the phenotype and genotype for crop N response and NUE. In an attempt to identify phenotypic traits for N-response and NUE in the earliest stages of plant growth, we analyzed the N-responsive germination, respiration, urease activities, and root/shoot growth of 21 Indica genotypes of rice (*Oryza sativa* var. *indica*). We found that N delays germination from 0 to 12 h in a genotype-dependent and source-dependent manner, especially with urea and nitrate. We identified contrasting groups of fast germinating genotypes such as Aditya, Nidhi, and Swarnadhan, which were also least delayed by N and slow germinating genotypes such as Panvel 1, Triguna, and Vikramarya, which were also most delayed by N. Oxygen uptake measurements in the seeds of contrasting genotypes revealed that they were affected by N source in accordance with germination rates, especially with urea. Germinating seeds were found to have endogenous urease activity, indicating the need to explore genotypic differences in the effective urea uptake and metabolism, which remain unexplored so far. Urea was found to significantly inhibit early root growth in all genotypes but not shoot growth. Field evaluation of 15 of the above genotypes clearly showed that germination rates, crop duration, and yield are linked to NUE. Slow germinating genotypes had longer crop duration and higher yield even at lower N, indicating their higher NUE, relative to fast germinating or short duration genotypes. Moreover, longer duration genotypes suffered lesser yield losses at reduced N levels as compared to short duration genotypes, which is also a measure of their NUE. Together, these results indicate the potential of germination rates, crop duration, urea utilization and its effect on root growth in the development of novel phenotypic traits for screening genotypes and crop improvement for NUE, at least in rice.

## Introduction

Nitrogen is quantitatively the most important nutrient input for intensive crop production, and the improvement of Nitrogen use efficiency (NUE) is an important economic and environmental goal (Hakeem et al., 2011; Sutton and Bleeker, 2013). While this is generally true for all crops, rice is particularly important, not only because it is the third most produced and consumed food grain in the world^1^, but also because of its lowest NUE among the cereals (Norton et al., 2015), consuming most of the cultivated land in India^2^ and about half of all N fertilizer used in Indian agriculture (Abrol et al., 2017). The identification of the biological approaches for improvement of fertilizer NUE is hampered by a lack of clarity on what constitutes the true phenotype and genotype for crop N response and NUE (Pathak et al., 2011; Sinha et al., 2018). There have been some attempts toward phenotypic characterization of the various traits associated with N response and NUE in rice such as, root length and density (Morita et al., 1988; Yang et al., 2012; Peng et al., 2015; Rogers and Benfey, 2015; Steffens and Rasmussen, 2016), dense and erect panicle (Sun et al., 2014) etc.

Seed germination is extremely important for vigor and crop performance (Rajjou et al., 2012). Oxygen consumption, which drives seed germination, is considered to affect biomass and therefore yield, as it uses up the photosynthetic reserves (Cannell and Thornley, 2000). On the other hand, considerable clarity was obtained on the role of oxygen consumption *vis-à-vis* yield; the mechanism involves oxygen consumption driven acceleration of the meristematic growth (starting with germination), which in turn hastens the life cycle such as branching, such that the plant matures faster and yield is lowered due to shortened life span of the photosynthetic plant (Sitaramam et al., 2008a). Thus, oxygen consumption affects yield through controlling the life cycle, starting with germination and branching upto flowering and yield. By osmotic titrations of the growth stages, it was shown that mitochondrial energetics via oxygen consumption regulate vegetative and reproductive branching, which determine the life span and yield in Arabidopsis (Sitaramam and Atre, 2007).

Studies on the effect of N supply on germination, on the other hand, have not been conclusive, and varied between no effect (Monaco et al., 2003; Çatav et al., 2015; Schnadelbach et al., 2016), N-enhanced germination in dicots (Srivastava and Chauhan, 1977; Pérez-Fernández et al., 2006; Al-Harbi et al., 2008; Zeng et al., 2015) to N-inhibited germination in monocots (Wan et al., 2016; Wen et al., 2017) including rice (Haden et al., 2011; Qi et al., 2012). Some of these differences could be due to the different N-forms used or prevailing under different soil conditions such as temperature, moisture, pH, microbial population etc. (Russell et al., 2002).

Root growth parameters have been associated with NUE (Xu et al., 2012; Li et al., 2015; Kiba and Krapp, 2016; Xie et al., 2017), but systematic evaluation of different rice genotypes with different forms/doses of N are lacking. Similarly, while urea uptake, urease activity, and urea metabolism in rice are known (Cao et al., 2010; Wang et al., 2012), the role of endogenous urease in N response and/or NUE of rice or any other cereal crop remains unexplored.

The overall aim of the present study was to identify early growth-related phenotypic traits for N-response and NUE by evaluating the effect of N, not merely on biomass or yield but on the critical physiological parameters of the plant life cycle, beginning with germination. For this purpose, we compared N-responsive changes in the germination rates of 21 rice genotypes using different N forms to rank them by N-response and validated 15 of them in the field for crop duration and yield, to identify contrasting genotypes and to analyze their seedling growth and urease activities.

## Materials and Methods

### Plant Materials

Twenty-one rice genotypes of *Oryza sativa* L. ssp. *indica* were used in this study, spanning 11 out of 15 agro-climatic zones and various soil types of India, except the Western Himalayan, Western dry region, desert, and island regions. They also span a range of crop durations (92–150 days) and yields (3.6–7.8 t/ha). Of these 21, the seeds of 20 genotypes were procured from the Indian Institute of Rice Research, Hyderabad, India. They were, Aditya, Swarnadhan, Nidhi, Jaya, Vikas, Ajaya, Krishnahamsa, Mandyavijaya, Nagarjuna, Prasanna, Pusa Basmati, Ravi, Rasi, Sampada, Suraksha, Swarna, Triguna, Varadhan, Vibhava, and Vikramarya. Seeds of the genotype Panvel 1 were procured from Kharland rice research station, Panvel, Maharashtra, India. All the 21 genotypes were used for germination studies in the lab, following which contrasting pairs were identified and used for oxygen consumption, growth, and urease activity measurements. Independently, 15 of these genotypes were evaluated in the field at the Indian Institute of Rice Research, Hyderabad, as detailed under “Field experimental conditions” (see below).

### Laboratory Growth Conditions

Seeds of the selected rice genotypes were weighed individually and only seeds of modal weight were used as described earlier (Sitaramam et al., 2008a) for all experiments. They were surface-sterilized with 0.1% mercuric chloride for 50 s followed by 8–10 washes with double distilled water. They were then soaked in distilled water for 2 h and plated on UV-sterilized moist cotton in 120 mM Petri plates containing water or Arnon Hoagland media (Hoagland and Arnon, 1950) with or without nitrate/urea/ammonia/ammonium nitrate as the sole nitrogen source. The chemicals used to make Arnon Hoagland media were obtained from SRL, India. The normal source of N in the Arnon Hoagland medium was nitrate, in the form of KNO_3_ (5 mM) and Ca (NO_3_)_2_ (5 mM), which were replaced with urea (7.5 mM), or NH_4_NO_3_ (7.5 mM), or NH_4_Cl (15 mM) as the sole N source, at an equalized concentration of 15 mM. Distilled water and N-free media were used as controls. The Petri plates were incubated in a plant growth chamber at 28°C, 75% humidity, 600 lux light intensity at the plant level, obtained from Osram fluorescent tubes and 12 h/12 h photoperiod.

### Field Experimental Conditions

Fifteen of the above mentioned Indica rice genotypes were used for field evaluation, namely Aditya, Swarnadhan, Rasi, Jaya, Varadhan, Ravi, Swarna, Suraksha, Vibhava, Vikas, Krishna Hamsa, Sampada, Prasanna, Pusa Basmati, and Triguna. This evaluation was conducted at the farm of the Indian Institute of Rice Research, Hyderabad, India, for six seasons over 3 years (two seasons per year, Kharif and Rabi) from 2010–2011 to 2014–2015. The geographical coordinates of the experimental farm are, 17°19″ N latitude and 78°23″ E longitude, at an altitude of 542 m above sea level and mean annual precipitation of 750 mm. The characteristics of field soil was pH 8.1; EC 0.7l dS/m; free CaCO_3_ 5.01%; CEC 44.1 C mol (p+)/kg soil; soil organic carbon 0.70%; Soil available N 215 kg/ha; available phosphorus 46 kg P/ha; potassium 442 kg K/ha, and zinc 12.5 ppm.

The field experiment was based on a split plot design of plot size 7.0 m × 3.5 m with three replicates. Urea was the sole source of N, applied at the rate of 100 kg N/ha (N100) or 217 kg urea/ha in three equal splits (1/3 at basal, 1/3 at tillering, and 1/3 at panicle initiation stage), with a control of no added N (N0). To maintain isolated conditions between plots, the field was divided into two separate blocks of N-0 and N-100 by making a deep trench of 4 feet between them and placing thick polythene sheets in the trench deep into the soil to avoid leaching from plot to plot and these plots were being maintained permanently. Moreover, the same exact plots were used for N0 and N100 throughout the six seasons. Yield and crop duration was measured using a sample of one meter square from two different areas of each plot, containing approximately 66 plants per sample.

### Germination and Oxygen Consumption Measurements

The number of seeds germinated in each plate of 100 seeds in triplicate (300 seeds per treatment) was monitored every 3 h in terms of the visible emergence of the radical, until all the seeds in the plate visibly germinated. The germination rate is defined as the time taken for 50% seeds to germinate (t_½_). It was calculated by interpolating the data using the X_0_ function in SigmaPlot software version 9. Oxygen consumption was measured as the rate of oxygen consumption at the t_½_ time point for selected contrasting rice genotypes using Oxygraph 2.1 from Hansatech, United Kingdom. For this purpose, four seeds each of modal weight were taken in duplicates in cuvettes filled with media with or without nitrate/urea, and O_2_ consumption was monitored for 12 min for every measurement.

### Shoot/Root Length Measurement

Ten seeds each of the selected rice genotypes were placed in vertically positioned Petri plates half-filled with 0.8% plant agar prepared in Arnon Hoagland media with or without nitrogen in triplicate. Shoot/root length was recorded in centimeter on the seventh day by using software Image J. The entire experiment was repeated thrice with three technical replicates (10 seeds × 3 technical × 3 biological replicates per treatment).

### Urease Assay

Qualitative urease assay was performed by phenol red indicator method using germinating seeds of the genotype Panvel 1. For this purpose, triplicate sets of 100 seeds each were soaked in autoclaved deionized water for 2 h and plated on Arnon Hoagland media with or without urea as the sole N source. At 72 h, when two-thirds of them showed visible signs of germination, they were qualitatively tested for urease using either intact or crushed seeds in an assay mixture that contained urea broth and Arnon Hoagland media in equal proportions (with or without urea as the sole N source). The media in which the seeds were grown were also tested to ensure that there was no urease activity due to microbial contamination during the experiment. As a positive control, garden soil sample was tested and change of color was observed. Quantitative estimation of endogenous urease activity was done by spectrophotometric detection of ammonium ions released, as described by Kayastha et al. (1995) with two contrasting groups of rice genotypes namely Aditya/Nidhi and Panvel 1/Vikramarya.

### Data Analysis

SigmaPlot software ver. 9.0 was used to calculate t_½_ or the time taken for 50% seeds to germinate [Y = a/1 + exp^(X-X_0_)^b], Image J was used to measure shoot/root length, SPSS ver. 16 was used to perform two-way classified ANOVA for the significance of t_½_ and graphs were plotted using MS Excel software. Biplot analysis of the principal components was carried out by singular value decomposition method of the matrix by using XLispStat software (Udina, 2005; Parsad et al., 2007).

## Results

### Rate of Germination Varies With Genotypes

**FIGURE 1:**
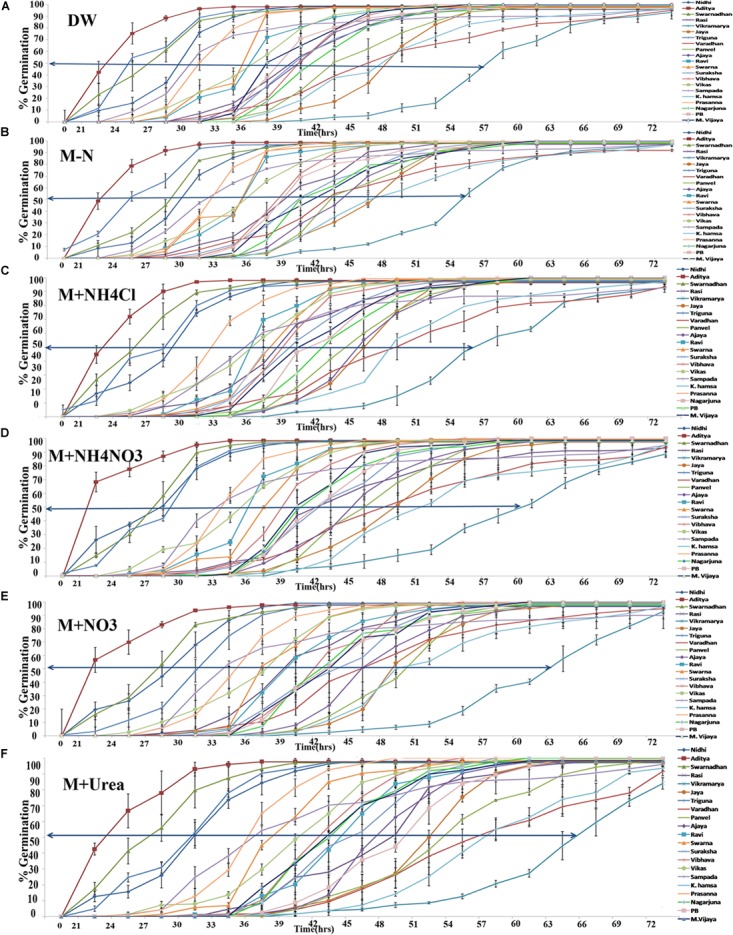
Variation in the germination rates among 21 rice genotypes with or without N. One hundred seeds of each of the 21 genotypes were plated in triplicate (300 seeds per treatment) on moist cotton and soaked with Arnon Hoagland medium with or without nitrate, ammonium, or urea as the sole source of N. Percent germination in each plate was monitored every 3 h until all the seeds have germinated and mean % values were plotted. Each curve represents a separate genotype and each graph corresponds to a different N regime such as distilled water **(A)**, media without N **(B)**, media with ammonium chloride **(C)**, or ammonium nitrate **(D),** or calcium and potassium nitrate **(E)**, or urea **(F)**. The arrows represent the time taken for 50% seeds to germinate (t_½_) deduced using X_0_ function of SigmaPlot ver. 9.

The time course of germination in different genotypes of rice was measured in triplicate by monitoring the % seeds germinated on moist cotton every 3 h until all the 100 seeds in the plates had visibly germinated. Germination curves were plotted for all the 21 genotypes in distilled water (A), Arnon Hoagland media without any N source (B) or media with ammonium chloride (C), ammonium nitrate (D), potassium and calcium nitrate (E) or urea (F) as the sole source of nitrogen (**Figure 1**). Individual germination curves for each of the 21 genotypes are provided as **Supplementary Figures 1–21**. It is evident that these are typically sigmoid curves of the kind [Y = a/1 + exp^(X-X_0_)^b], symmetric around the central value,without any visual aberrations. Therefore, X_0_, the mid-point across which the curve is symmetric (shown as arrows in **Figure 1**), represents t_½_ or the time taken for 50% seeds to germinate. This time point was used as a relevant coefficient to compare, distinguish and choose between genotypes and treatments. The coefficients were significant for all N treatments (*P* < 0.05, ANOVA). The data revealed that firstly, the genotypes varied considerably in their inherent rates of germination, based on which, they were sorted into fast germinating and slow germinating genotypes. ANOVA (LSD) analysis revealed that this observation is significant (*P* < 0.05, ANOVA) (**Table 1**). For example, the genotype Aditya had the fastest germination curve while Vikramarya was the slowest in all the media. Secondly, the germination rates (t_½_) in distilled water and media without N were relatively much faster, whereas they were increasingly and significantly slower in the presence of N (*P* < 0.05, ANOVA). LSD analysis showed more significant N-induced delay of germination by nitrate (*P* < 0.01) or urea (*P* < 0.01) but the delay was less significant in the case of ammonium chloride or ammonium nitrate (*P* < 0.05) (**Table 1**). Thirdly, the N-induced delay in germination was best resolved at t_½_ or the time taken for 50% seeds to germinate (shown as arrows in **Figure 1**).

**Table 1:** Analysis of variance of germination rates of 21 rice genotypes under different N regimes.

	DW	M-N	M+NH_4_Cl	M+NH_4_NO_3_	M+NO_3_^-^	M+Urea	Mean
Aditya	21.553	20.986	21.499	18.321	20.314	22.015	20.781**^n^**
Ajaya	41.751	42.265	44.716	43.742	45.486	46.337	44.049**^e^**
Jaya	46.396	44.760	44.782	47.325	47.950	50.560	46.962**^c^**
K. Hamsa	45.127	45.257	47.581	48.455	48.799	56.350	48.594**^b^**
MandyaVijaya	37.154	40.274	39.829	39.390	41.910	41.822	40.063**^g^**
Nagarjuna	40.264	40.000	40.725	39.892	41.375	42.114	40.728**^gf^**
Nidhi	27.912	28.116	27.224	25.680	27.281	29.743	27.659**^l^**
Panvel 1	42.833	42.640	43.507	44.970	48.206	52.279	45.739**^d^**
Prasanna	30.030	30.971	31.363	32.282	33.577	34.863	32.180**^k^**
Pusa Basmati	37.223	37.454	41.748	40.921	41.929	48.101	41.229**^f^**
Rasi	32.717	37.817	37.182	41.711	38.342	46.288	39.009**^h^**
Ravi	31.420	30.336	32.020	31.554	35.416	39.799	33.424**^j^**
Sampada	28.964	30.462	33.854	31.580	32.198	35.158	32.036**^k^**
Suraksha	38.887	39.361	38.569	41.415	42.219	43.818	40.711**^gf^**
Swarna	32.231	32.738	36.651	36.136	36.604	37.178	35.256**^i^**
Swarnadhan	24.929	26.906	24.003	25.760	26.166	25.577	25.556**^m^**
Triguna	24.505	24.203	25.985	26.852	30.374	28.995	26.819**^l^**
Varadhan	44.530	41.285	46.483	45.578	44.448	53.846	46.028**^d^**
Vibhava	39.020	37.012	37.791	37.806	41.077	41.295	39.00**^h^**
Vikas	34.429	33.789	34.723	33.761	35.560	39.256	35.253**^i^**
Vikramarya	55.447	52.937	55.167	58.419	62.214	66.899	58.513**^a^**
Mean	36.063**^d^**	36.170**^d^**	37.400**^c^**	37.692**^c^**	39.116**^b^**	42.013**^a^**	
	LSD at 5% for treatment			0.486			
	LSD at 5% for genotype			0.909			
	LSD at 5% for treatment ^∗^ genotype			2.227			


*One hundred seeds of each of the 21 genotypes were plated in triplicate (300 seeds per treatment) on moist cotton and soaked with distilled water, Arnon Hoagland medium with or without nitrate, ammonium, or urea as the sole source of N. Percent germination in each plate was monitored every 3 h until all the seeds have germinated and the time taken for 50% seeds to germinate (t_½_) were deduced for each replicate under different N regimes using X_0_ function in SigmaPlot ver. 9 and subjected to two-way classified ANOVA. Mean values for all genotypes and all N treatments are categorized into statistically distinct classes as denoted by their superscripted alphabetic character. Values with different alphabets are significant at P < 0.05.*

### N Delays Germination in a Genotype-Dependent and N-Source-Dependent Manner

**FIGURE 2:**
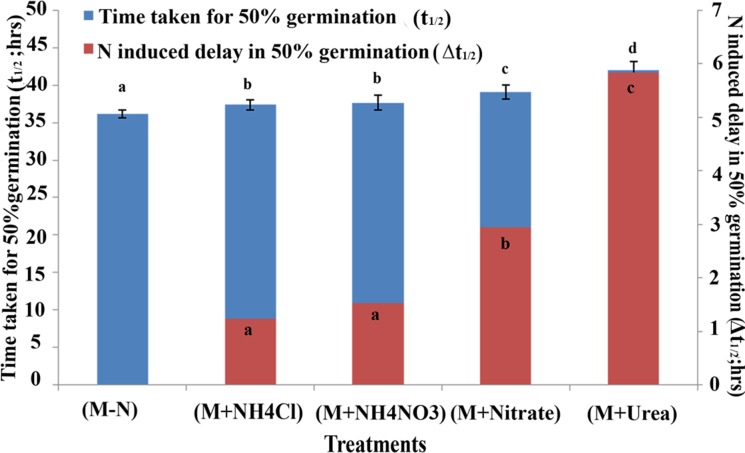
Effect of N on germination at the mean time taken for 50% seeds to germinate among 21 Indica rice genotypes. The mean time taken for 50% seeds to germinate (t_½_) in Arnon Hoagland media with or without N for all the 21 genotypes were plotted for each treatment, along with their standrad error values (error bars at Y1 axis). The extent of delay in germination caused by each N source relative to that observed in media without N is shown as an inset in each bar using an expanded scale on the Y2 axis. The data are categorized into statistically distinct classes as denoted by alphabetic character for each set of bars (red and blue). Bars with different alphabets are significant at *P* < 0.05.

A comparison of all the genotypes at the t_½_ time point is shown in **Table 1**, which highlights the variation in the germination rates between the genotypes ranging between 22 to 55 h in distilled water, 21 to 53 h in media without N, 21 to 55 h in media with ammonium chloride, 18 to 58 h with ammonium nitrate, 22 to 62 h with potassium and calcium nitrate and 22 to 67 h with urea. These data clearly show that the germination rate of each genotype varied uniquely in response to each form of N in the media, which was also best resolved and quantified in terms of the delay in the time taken for 50% seeds to germinate (t_½_). The effect of N treatment on germination rate was also captured in terms of the mean t_½_ values of all genotypes as shown in **Figure 2**. It revealed that N-induced delay in germination was true for all genotypes, varying only in magnitude depending on the source of N and the genotype. This time difference between the t_½_ values in the media with and without N was termed as Δt_½_. The average extent of delay in germination caused by each N source relative to media without N (Δt_½_) for all genotypes is shown as an inset in each bar using an expanded scale on the Y2 axis of **Figure 2**. Ammonium chloride caused the least delay in the time t_½_ (by 1 h 30 min), followed by the increasingly delaying effect of ammonium nitrate (1 h 48 min), nitrate alone (3 h) and urea, which had the highest delaying effect of 6 h on t_½_. While these are average values for all genotypes, the actual extent of N-induced delay varied considerably among genotypes.

### Genotypes Can Be Ranked Based on Inherent or N-Responsive Germination Rates

All the 21 genotypes used in this study were ranked based on the time taken for 50% seeds to germinate (t_½_) as an inherent property of each genotype in any given medium, as well as on the basis of the extent of N-induced delay in germination relative to media without N (Δt_½_). The first ranking identified fast-germinating and slow-germinating genotypes in each medium (**Table 2**), whereas the second ranking identified the genotypes as least N-responsive or most N-responsive, based on whether germination rates were least affected or most affected by a given source of N (**Table 3**). Interestingly, both types of rankings coincided to a great extent, with the genotypes Aditya, Swarnadhan, and Nidhi occupying the top ranks and Panvel 1, Triguna, and Vikramarya faring toward the bottom in both **Tables 2, 3**, especially with respect to nitrate and urea treatments. These trends were not very consistent or significant with ammonium chloride as the N source. The statistical validity of ranking by t_½_ as well as Δt_½_ was confirmed by Kendall’s tau_b correlation coefficients, which were found to be significant at 0.01 level, except in the case of Δt_½_ ranking for ammonium chloride treatment (**Supplementary Table 1**).

**Table 2:** Ranking of 21 Indica rice genotypes based on germination rate.

Genotypes	DW	(M-N)	(M+NH_4_Cl)	(M+NH_4_NO_3_)	(M+NO_3_)	(M+Urea)
Aditya	1	1	1	1	1	1
Swarnadhan	2	3	2	3	2	2
Nidhi	4	4	4	2	3	4
Triguna	3	2	3	4	4	3
Sampada	9	5	6	5	5	6
Prasanna	5	6	5	6	6	5
Vikas	8	9	7	7	7	8
Swarna	7	7	8	9	8	7
Ravi	6	8	19	8	9	12
Vibhava	13	10	9	10	10	9
Rasi	15	15	12	15	11	15
Nagarjuna	14	13	13	12	12	11
MandyaVijaya	10	14	11	11	13	10
Pusa Basmati	11	11	14	13	14	14
Suraksha	12	12	10	12	15	13
Varadhan	17	16	17	17	16	19
Jaya	20	18	16	19	17	17
Panvel	16	17	15	16	18	18
Ajaya	18	19	20	18	19	16
K. Hamsa	19	20	18	20	20	20
Vikramarya	21	21	21	21	21	21


*The time taken for 50% seeds to germinate (t_½_) was computed from germination profiles generated from triplicate plates with 100 seeds each sown on moist cotton containing distilled water (DW) or Arnon Hoagland media (M) with or without N as specified in the titles of the columns. The ranking was based on the t_½_ values obtained in medium with nitrate (first column), which corresponds well with other N treatments.*

**Table 3:** Ranking of 21 Indica rice genotypes based on the extent of N-induced delay in germination.

	Rank on the basis of Δ t_½_			
	
Genotypes	ΔM±NO_3_	ΔM±NH_4_Cl	ΔM±Urea	Δ(M±NH_4_NO_3_)
Aditya	1	18	2	1
Nidhi	2	4	4	2
Swarnadhan	3	14	1	3
Rasi	4	7	15	16
Nagarjuna	5	19	5	5
MandyaVijaya	6	11	3	4
Sampada	7	17	11	8
Vikas	8	5	13	6
Prasanna	9	3	6	10
Suraksha	10	12	10	12
Varadhan	11	1	20	20
Ajaya	12	9	7	11
Jaya	13	15	14	14
K. Hamsa	14	10	19	17
Swarna	15	20	9	18
Vibhava	16	16	8	7
Pusa Basmati	17	13	18	19
Ravi	18	21	16	9
Panvel	19	8	17	13
Triguna	20	2	12	15
Vikramarya	21	6	21	21


*The N-induced delay in the time taken for 50% seeds to germinate (Δt_½_) was calculated as the difference between t_½_ values in Arnon Hoagland media (M) with or without N, as specified for each source of N in the titles of the columns. The ranking was based on the values obtained in media containing nitrate (first column).*

### Slow Germinating Genotypes Are Most N-Responsive *vis-à-vis* Fast Germinating Genotypes

**FIGURE 3:**
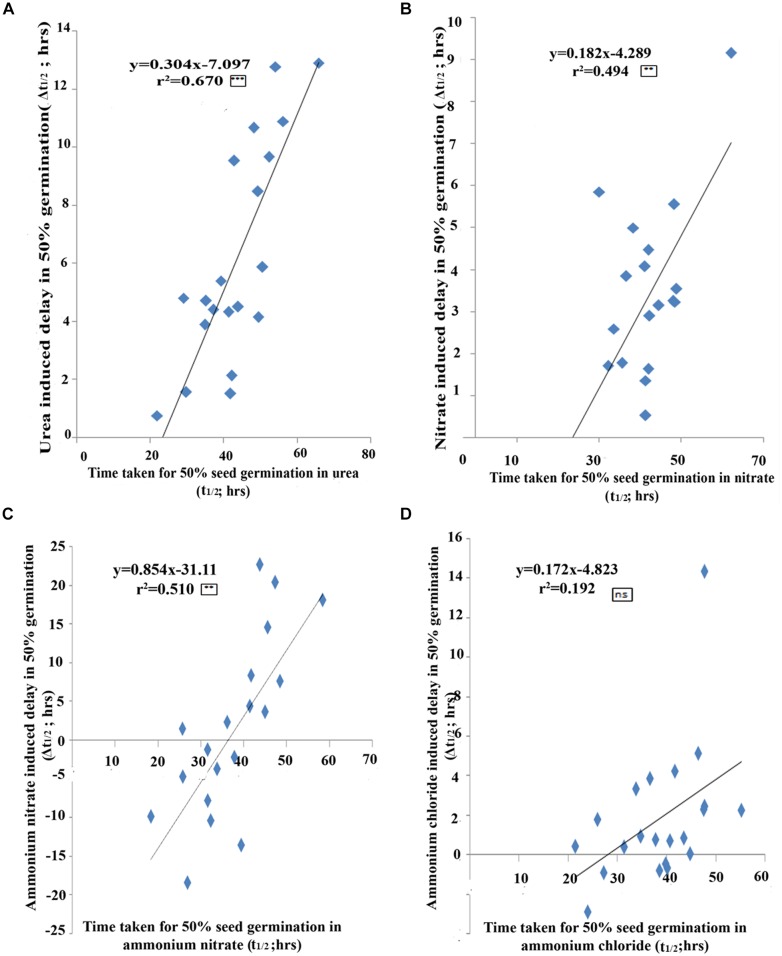
The extent of N-effect on germination as a function of the inherent germination rates of genotypes. Scatter plot of 21 genotypes between the time taken for 50% seeds to germinate (t_1/2_) and N-induced delay in 50% seeds to germinate (Δt_1/2_) for urea **(A)**, nitrate **(B)**, ammonium nitrate **(C)**, and ammonium chloride **(D)** N treatments. The effect of N increases with the inherent germination rate of the genotype, with fast germinating genotypes least delayed by N whereas slow germinating genotypes are most delayed by N. The significance levels are shown as stars next to *R*^2^ values. ^∗^Denotes significant at *P* < 0.05, ^∗∗^denotes significant at *P* < 0.01, ^∗∗∗^denotes significant at *P* < 0.001 and ns denotes non-significant.

A statistical validation of the above relationship between the t_½_ and Δt_½_ values among genotypes revealed a significant correlation for urea (*r*^2^ = 0.670; *P* < 0.001, **Figure 3A**) as well as for nitrate (*r*^2^ = 0.494; *P* < 0.01, **Figure 3B**), ammonium nitrate (*r*^2^ = 0.5102; *P* < 0.01, **Figure 3C**), but not significant for ammonium chloride treatments (*r*^2^ = 0.1921; 3D). In other words, the fast germinating genotypes were least delayed by urea/nitrate N treatment or were least N-responsive, while slow germinating genotypes were most delayed by N and were most N-responsive. This statistically validated the ranking of genotypes by t_½_ and Δt_½_ in **Tables 2, 3** and the identification of the contrasting groups on that basis. The fast germinating genotypes Aditya, Swarnadhan, and Nidhi were least responsive to N, whereas the slow germinating genotypes Panvel 1, Triguna, and Vikramarya were most responsive to N.

### Biplot Analysis Validates Contrasting Genotypes for N-Response

**FIGURE 4:**
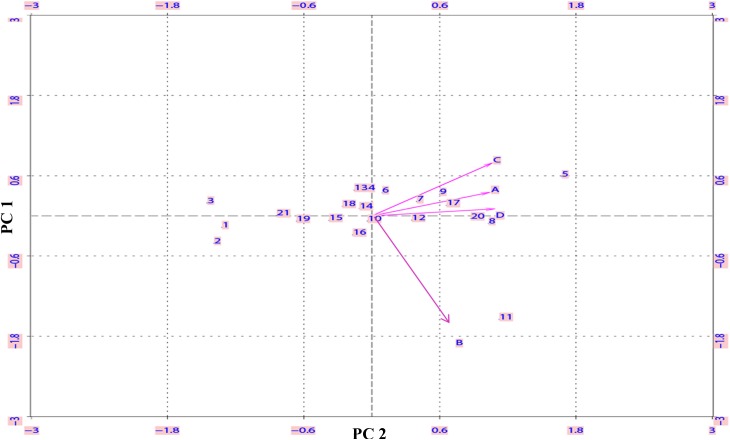
Biplot analysis of 21 Indica rice genotypes based on the extent of N-induced delay in germination. The N-responsive delay in the time taken for 50% seeds to germinate (Δt_½_) was calculated as the difference between the t_½_ values with or without N in the medium (as described in the legend for **Figure 1**) and subjected to biplot analysis. The numbers inside the plot represent the 21 genotypes and alphabets represent N treatments viz, nitrate (A), ammonium chloride (B), urea (C), or ammonium nitrate (D). The genotypes toward negative *X* axis are, Aditya (1), Nidhi (2), Swarnadhan (3), Mandya Vijaya (19) and Nagarjuna (21) whereas genotypes toward positive *X* axis are, Vikramarya (5), Varadhan (8), Panvel (9) and Pusa Basmati (20).

To statistically validate the contrasting N-response among genotypes under different N-treatments, biplot analysis was done on Δt_½_ values by singular value decomposition method using XLispStat software. The plot shown in **Figure 4** allowed the identification of the most varied genotypes that accounted for most of the phenotypic variance in N response. The genotypes fell into three clusters: Aditya, Nidhi, Swarnadhan, Mandyavijaya, and Nagarjuna lie at the farthest end of the negative *X* axis, while Panvel 1, Vikramarya, Vardhan, and Pusa Basmati clustered toward the farthest end of the positive *X* axis, and leaving the rest of the genotypes clustering around the center between the above two clusters. All the N treatments except ammonium chloride clustered together, validating their similar effect on delayed germination.

### Field Data Reveal Significant Relationship Between Crop Duration and Yield

**FIGURE 5:**
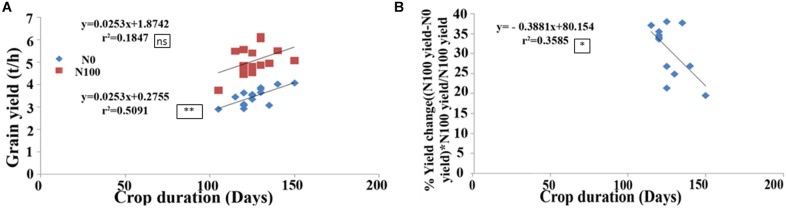
Correlation between crop duration and grain yield in the field. Crop duration and grain yield of 15 Indica rice genotypes was measured in the field without added N (N0) or with 100 kg/ha urea N (N100) for 6 seasons over 3 years, and their mean values were used for correlation analysis. **(A)** Correlation between crop duration and total grain yield was significant in N0 but not in N100. **(B)** Negative correlation between crop duration and % yield change or loss in N0, relative to N100 [(N100 yield–N0 yield)^∗^N100 yield/N100 yield], indicating higher N use efficiency at longer crop duration. The significance levels are shown as stars next to *R*^2^ values. ^∗^Denotes significant at *P* < 0.05, ^∗∗^denotes significant at *P* < 0.01, and ns denotes non-significant.

Field performance of 15 of the above genotypes was evaluated with 100 kg/ha urea-N (N100) or without added N (N0) over six seasons spanning 3 years as described in the Section “Materials and Methods” and their crop duration and yield was recorded (**Supplementary Table 2**). These two traits revealed significant correlation under N0 condition (*r*^2^ = 0.509, *P* < 0.01, **Figure 5A**), but not under N100 condition, despite similar trend (**Figure 5A**). The most important outcome of this relationship was that the longer duration genotypes Varadhan, Pusa Basmati, Krishnahamsa, and Triguna (which were slow germinating and most responsive to N treatment, as their germination was most delayed by N in the lab) gave significantly higher yield in the field under N0 condition, indicating higher NUE, as compared to the short duration genotypes Aditya, Swarnadhan, and Rasi, whose germination was least delayed by N (**Figure 5A**). More importantly, crop duration was significantly negatively correlated with % yield change or loss at N0 in comparison to N100 (*r*^2^ = 0.358 at *P* < 0.05, **Figure 5B**). The % yield change was calculated as (N100 yield-N0 yield)^∗^(N100 yield)/N100 yield. This means that long duration genotypes suffered less yield penalty under N0 in comparison to N100, indicating their higher NUE than short duration genotypes.

### Germination Rates Are a Function of Oxygen Consumption Rates

**FIGURE 6:**
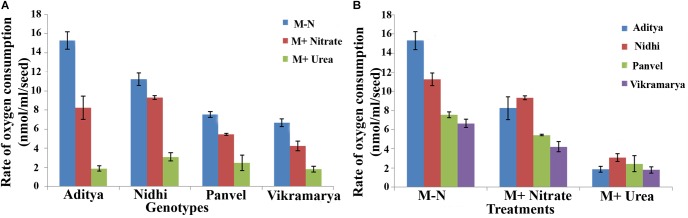
Oxygen consumption rates among genotypes with contrasting rates of germination with or without N. Rates of oxygen consumption were measured in duplicates using oxygen electrode for two contrasting pairs of rice genotypes at the time taken for 50% seeds to germinate (t_½_) and shown in terms of genotypes **(A)** or treatments **(B)**. Their significance level at *P* < 0.05 has been shown as alphabets in **Table 4**.

In order to investigate whether the differences in N-responsive germination rates (Δt_½_) are a reflection of differences in oxygen consumption, oxygen uptake measurements were done using four contrasting genotypes. They are, Aditya and Nidhi from the top-ranking group and Panvel 1 and Vikramarya from the bottom ranking group in **Tables 2, 3**. Four germinated seeds of each genotype were used at its t_½_ with or without N for O_2_ consumption measurement in duplicates. The number of seeds used for the experiment was standardized based on the maximum seeds that could rotate freely in the cuvette. **Figure 6** shows the mean O_2_ consumption values arranged genotype-wise (6A) or treatment-wise (6B). The genotypes showed decreasing oxygen consumption in line with their ranking, meaning that top ranking genotypes that germinate fast showed higher rates of oxygen consumption, whereas the bottom ranking genotypes that germinate slow showed lower rates of oxygen consumption (**Figure 6A**). In other words, faster the germination, faster the oxygen consumption. Student’s *t*-test revealed that independent of N, the difference in oxygen consumption between fast and slow germinating genotypes is significant (*P* < 0.05). Furthermore, there was significant inhibitory efffect of N on oxygen consumption, not only in all genotypes taken together (*P* ≤ 0.05 in nitrate and *P* ≤ 0.01 in urea), but also in fast or slow subsets (*P* < 0.05 in nitrate and *P* < 0.01 in urea), except in Vikramarya. The ANOVA analysis of differences in oxygen consumption between various treatments and genotypes is provided separately in **Table 4**.

**Table 4:** Oxygen consumption rates among genotypes with contrasting rates of germination with or without N.

Genotype∖Treatment	M-Nitrogen	M+Nitrate	M+Urea	Genotype means over treatments
Aditya	15.29	8.25	1.87	8.47^a^
Nidhi	11.25	9.33	3.08	7.88^a^
Panvel	7.54	5.45	2.46	5.15^b^
Vikramarya	6.67	4.23	1.81	4.23^b^
Treatment means over genotypes	10.18^a^	6.81^b^	2.30^c^	6.43
	LSD at 5% for Genotype = 1.2463			
	LSD at 5% for Treatments = 1.0793			
	LSD at 5% for Genotype × Treatment interaction = 2.1586			


*Rates of oxygen consumption were measured in duplicates using oxygen electrode for two contrasting pairs of rice genotypes at the time taken for 50% seeds to germinate (t_½_) and shown in terms of genotypes and treatments. Mean values for all genotypes and all N treatments are categorized into statistically distinct classes as denoted by their superscripted alphabetic character. Values with different alphabets are significant at P < 0.05.*

When compared in terms of the effect of N source, all genotypes showed moderately reduced oxygen consumption in the presence of nitrate as compared to media without N, whereas the oxygen consumption was highly diminished in the presence of urea in all genotypes (**Figure 6B**). Despite these differences due to variations in the O_2_ uptake values found in individual genotypes, the fact remains that both nitrate and urea have significant overall inhibitory effects on oxygen consumption on all genotypes put together, in line with their inhibitory effects on germination (**Table 4**).

### Germinating Seeds Have Endogenous Urease Activity

**FIGURE 7:**
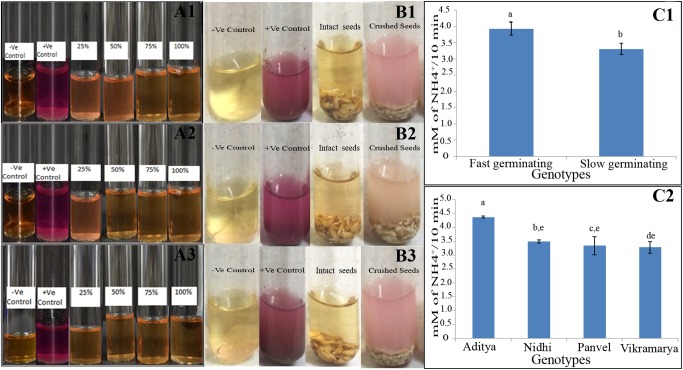
Urease qualitative assay in the germination media **(A)** and germinating seeds of one genotype **(B)** and quantitative assay in contrasting genotypes **(C)**. For **A**, the Arnon Hoagland media from Petriplates having 25, 50, 75, and 100% germinated seeds of the genotype Panvel 1 were qualititively assayed in triplicates **(A1–A3)** in urea broth for external urease, using soil as the positive control and only urea broth as the negative control. For **B**, all the 66 germinated seeds from two-thirds germinated plate were transferred either intact or after crushing, to test tubes containing Arnon Hoagland media and phenol red for qualitative assay of urease in triplicates **(B1–B3)**. For **C**, quantitative urease assays were performed using the extracts of germinating seeds of fast germinating genotypes Aditya and Nidhi to contrast them with slow germinating genotypes, Panvel and Vikramarya. **C1** shows the mean urease activities between fast and slow germinating genotypes, whereas **C2** shows the activities in individual genotypes. The data are categorized into statistically distinct classes as denoted by alphabetic characters above the bars. Bars with different alphabets are significantly different at *P* < 0.05.

In order to investigate whether the strong effect of urea on germination and oxygen consumption depends on urea metabolism within the seed or in the external environment, urease activity was initially assayed qualitatively using the seeds of the genotype Panvel 1 and later quantitatively by comparing two contrasting pairs of genotypes namely Aditya, Nidhi from the top-ranking group and Panvel and Vikramarya from the bottom-ranking group in triplicates. The data in **Figure 7A** clearly shows that the Arnon Hoagland media used to germinate seeds in the Petri plates did not have any detectable urease activity throughout the 3 days taken for complete germination. This was also true for intact seeds assayed after completion of germination, whereas crushed seeds clearly showed urease activity, both in qualitative (**Figure 7B**) and quantitative assays (**Figure 7C**). This ruled out any contribution of microbial urease to the observed seed response to urea in terms of germination or oxygen consumption. The endogenous urease activity in even 2 g of crushed germinating seeds was far lower than that in a few milligram of soil in all four genotypes and is probably inadequate to account for all the observed effects of urea on respiration and germination. Therefore, the inhibitory effects of urea on respiration and germination are more likely to be direct effects of urea *per se* and not its metabolite. Interestingly, endogenous urease activities differed between contrasting pairs of genotypes, with significantly lower levels in the slow germinating genotypes (by 18.5%) as compared to fast germinating genotypes, despite internal variation (*P* < 0.05, **Figures 7C1,C2**).This merits further examination, to verify whether the differences in endogenous urease activities contribute significantly to the differences in NUE between genotypes.

### Urea Inhibits Root Growth More Significantly Than Shoot Growth

**FIGURE 8:**
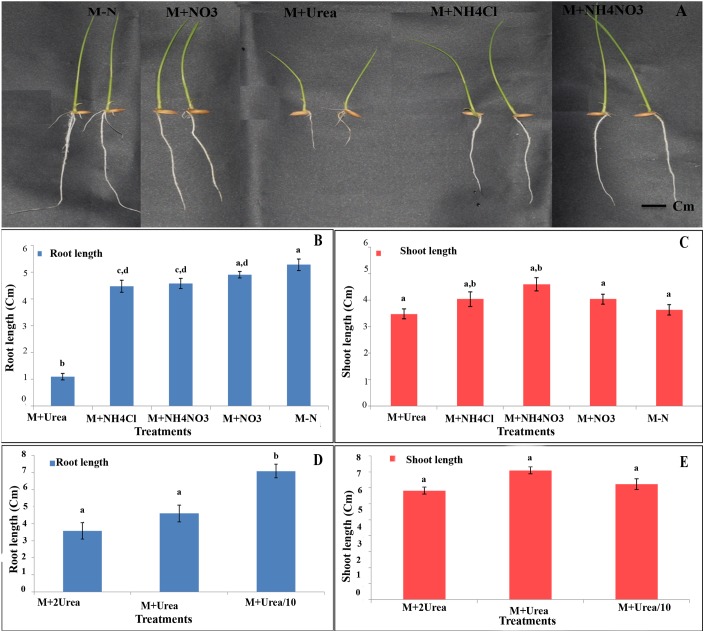
Effect of N on root/shoot length among contrasting rice genotypes. **(A)** Representative photograph of the seedlings of genotype Panvel 1 grown on agar plates in Arnon Hoagland media with or without N source. **(B,C)** Mean root lengths **(B)** and shoot lengths **(C)** of triplicates of 6 contrasting genotypes viz, Aditya, Nidhi, Swarnadhan from the fast germinating group and Panvel 1, Triguna and Vikramarya from the slow germinating group, in agar media with or without N. **(D,E)** The same genotypes were used to compare the effect of urea at different doses on root length **(D)** and shoot length **(E)**. The urea doses used were, normal level (15 mM), twice the normal (30 mM), and one-tenth (1.5 mM). The data are categorized into statistically distinct classes as denoted by alphabetic characters above the bars. Bars with different alphabets are significantly different at *P* < 0.05.

In order to explore whether the effect of N source on germination continues through the early development of the seedlings, their root and shoot lengths were measured in the six contrasting genotypes and their mean data are shown in **Figure 8**. There was little or no effect of any source of N on shoot length in any or all genotypes, except the significant increase in shoot length with ammonium nitrate (*P* < 0.01, ANOVA, **Figure 8C**). On the other hand, all the different N sources used have resulted in significantly reduced root lengths as compared to media without N (**Figure 8B**), except nitrate, for which the effect was not significant. At the normal levels of N, urea suppressed root length drastically (*P* < 0.01, ANOVA), followed by ammonia and ammonium nitrate (**Figure 8B**). The suppressive effect of urea was significantly higher when its concentration was doubled and was significantly lower when urea concentration was reduced to one-tenth, indicating a typical dose–response relationship (**Figure 8D**), which was not found in the case of shoot length (**Figure 8E**). Interestingly, though the suppressive effect of urea on root length was higher in fast germinating genotypes and relatively lower in slow germinating genotypes when taken together, these differences were not significant in individual genotypes, except in the genotype Panvel 1. This indicates that while the differential inhibition of root length by urea in individual genotypes needs further confirmation, the differences may not be as sharp as those seen in germination and oxygen consumption.

## Discussion

The lack of a clearly defined phenotype has been a major challenge for crop improvement for NUE, as is the lack of breeding programs for screening crop performance under low N input conditions. Plant N response has been traditionally characterized in terms of the morphological, physiological, or gene expression changes as a function of dose or form of N, such as nitrate, ammonium or both, or urea (Pathak et al., 2011; Gent and Forde, 2017; Mandal et al., 2018; Sinha et al., 2018). Out of the many parameters that may reflect N-response, including dose–response relationships between N input and yield, it is not yet clear how many measurable biological parameters specifically and significantly influence agronomic NUE (grain yield per unit N input) and to what extent. Some of the phenotypic characters reported to be associated with N-response/NUE so far include root growth (Xu et al., 2012; Li et al., 2015; Kiba and Krapp, 2016; Xie et al., 2017) and leaf chlorophyll content (Ladha et al., 2007; Thind and Gupta, 2010; Mathukia et al., 2014).

Germination, which is the most direct means to examine the meristematic behavior, has not been systematically examined as a phenotypic differentiator of any quantitative trait including NUE, despite the evidence that germination rate was tightly correlated with flowering time and yield in rice (Sitaramam et al., 2008a). Hence, the present study sought to explore N-responsive differences in germination and used them to identify contrasting rice genotypes that differed in N-responsive yield and therefore NUE.

The 21 genotypes used in our analysis span most of the Indian agro-climatic zones, soil types, diverse crop durations, and yields. Their germination curves revealed inherent variation between genotypes, allowing the identification of fast germinating and slow germinating genotypes (**Supplementary Figures 1–21**). The germination rates (t_½_) in distilled water and media without N were very similar for all genotypes, but N input caused delayed germination in a genotype-dependent and N-source-dependent manner (**Figure 1**). In other words, among all the components of Arnon Hoagland media, it is only N compounds that caused delay in germination. ANOVA (LSD) analysis clearly showed that the variation in germination rates between genotypes as well as between various N treatments was significant (**Table 1**). However, among various forms of N used, only nitrate and urea were highly significant in their effects on germination (*P* < 0.01), whereas ammonium chloride and ammonium nitrate were relatively less, though significant (P < 0.05, **Table 1**). As the pH of the media was adjusted to 5.8 in all the cases, it cannot be a reason for the different response of the seeds to ammonium salts. Volatilization of ammonia could be another reason, but since these differences did not contribute to statistically significant ranking of genotypes, it was not considered relevant to look deeper into it at this juncture.

In view of the clearly sigmoid nature of the germination curves, the time taken for 50% seeds to germinate (t_½_) was a competent metric to assess relative rates of germination (shown as arrows in **Figure 1**). A comparison of the mean t_½_ values of all genotypes under different N treatments clearly showed that while all N treatments caused delay in germination, urea delayed the most, followed by nitrate and ammonium salts (**Figure 2**). More detailed comparison of t_½_ values revealed genotype-wise differences in N-response (**Table 1**); fast germinating genotypes (in distilled water or media without N) were less delayed by N whereas the slow germinating genotypes were more delayed by N. These trends were also true in terms of N form, with urea causing the most delay in all the cases. This was further confirmed by the broad similarity in the ranking of genotypes based on their inherent t_½_ values in any given medium (**Table 2**) or on the extent of N-induced delay in the time taken for 50% seeds to germinate in each of the N forms (**Table 3**). This enabled the identification of contrasting genotypes such as Aditya, Nidhi, and Swarnadhan from the top rows of the above tables and Panvel 1, Triguna, and Vikramarya from the bottom rows. The relationship between t_½_ and Δt_½_ that formed the basis for such ranking was significant (**Figure 3**). Biplot analysis of the nature of variation between genotypes and treatments also revealed contrasting genotypes (**Figure 4**), providing independent statistical confirmation and thus validating most of those deduced manually from **Tables 2, 3**. The overall distribution of genotypes in **Figure 4** seems to follow the order of their Δt_½_ values from left to right, comparable to the top to bottom ranking in **Table 3**.

While the negative influence of N on rice germination was reported earlier in single genotypes (Haden et al., 2011; Qi et al., 2012), ours is the first systematic study based on energetics to demonstrate genotype-dependent variation in the magnitude of N-induced delay in germination and its utility to rank rice genotypes, using all three agronomically relevant forms of N, especially in rice. While all the parameters reported here are affected by N in some form or the other, different N forms affected different parameters differently. They could not have been reliably characterized in farm soil due to the microbial interconversions of various N forms before they enter the plant. Our experimental conditions relied on nutrient-free cotton regularly moistened with sterile media to rule out such microbial interconversions and specifically capture differential response of seeds to different forms of N.

Field evaluation of 15 of these above 21 genotypes for crop duration and yield (**Supplementary Table 2**) based on the availability of seeds and plots over six seasons under N0 and N100 (kg/ha of added N as urea) conditions revealed that the two traits are strongly correlated (**Figure 5A**). The higher yield in longer duration genotypes is especially significant under N0 condition (*r*^2^ = 0.509, *P* < 0.01, **Figure 5A**), an observation particularly relevant for NUE. Interestingly, the genotypes that had longer crop duration and higher yield in the field experiments were the slow germinating genotypes in the lab experiments. In terms of N response, the slow germinating genotypes are most N-responsive as in delayed germination in the lab (**Figure 3**). This presumably translates into extended crop duration and allows higher yield in the field (**Figure 5A**), even without any added N, by using residual N in the soil. Another interesting feature of the field data is the negative correlation between crop duration and % yield change or loss in N0 in comparison to N100 [(N100 yield–N0 yield)^∗^N100 yield/N100 yield]. In other words, longer duration genotypes suffer lesser yield loss under N0 relative to N100, indicating higher NUE at longer crop duration. This observation is significant at *P* < 0.05 (**Figure 5B**).

It must be emphasized that while the lab experiments on germination used totally N-depleted or N-replete media, the N0 condition in field experiments only indicates that there was no added N. The residual N in the soil depletes only gradually over 3 years of field experiments and therefore, N0 condition denotes low-N rather than zero-N. Nevertheless, considering that NUE is best assessed under low-N conditions, the finding that some genotypes gave higher yield under N0 than others, or suffered lesser yield loss under N0 than others, is significant from the NUE standpoint. We found that the longer duration genotypes Vardhan, Pusa Basmati, Krishnahamsa, and Triguna gave significantly higher yield in the field under N0 condition, indicating higher NUE, as compared to the short duration genotypes Aditya, Swarnadhan, and Rasi, whose germination was least delayed by N. More importantly, we identified at least 3 novel phenotypic traits associated with such NUE: time taken for 50% seeds to germinate (t_½_), the extent of N-induced delay in 50% seeds to germinate (or Δt_½_, a measure of N-responsiveness) and crop duration.

Germination is driven primarily by hydration and energy utilization and is a oxygen consumption-intensive phase in the development of a plant (Czarna et al., 2016). The role of nitrogen resources in enhancing plant productivity requires an explanation in terms of plant energetics, which remains unattempted so far. This could be due to the prevailing notion that oxygen consumption (photo oxygen consumption or otherwise) has an opposing influence on yield or is at least a confounding variable as it counteracts the conservation of the photosynthate exclusively toward yield. Sitaramam et al. (2008a) showed that meristem is a key determinant in the yield of rice, by demonstrating that the meristematic oxygen consumption hastens the life stages leading to less “time” available for accumulation of the photosynthate. They also showed that fast germinating rice genotypes have higher rates of oxygen consumption and mature faster with lesser grain yield, offering a testable paradigm of the role of energetics in plant yield, under which the role of nitrogen in the process could be studied. Therefore, we examined whether respiratory differences could account for delays in germination as well as for the effect of N on germination, using two contrasting pairs of genotypes. Indeed, the fast germinating (short duration) genotypes showed higher levels of O_2_ uptake as compared to slow germinating (long duration) genotypes (**Figure 6A**), while N input inhibited O_2_ uptake. Urea inhibited O_2_ uptake the most, while it was only moderately inhibited by nitrate, as compared to media without N (**Figure 6B**). These effects were more significant with urea rather than with nitrate, and the degree of inhibition between fast and slow genotypes did not reveal any contrasting pattern (**Table 4**). Nevertheless, it is clear that the observed differences in the germination rates between contrasting genotypes can be accounted largely by their oxygen consumption and additionally by the influence of N on oxygen consumption, germination, or both.

As the seeds were germinated in Petri plates on cotton moistened with sterilized media, the inhibitory effect of N in the form of urea is expected to be a chemical effect of urea uptake rather than a metabolic effect, unlike farm soil, in which microbial urease activity plays a major role. This was confirmed by the lack of detectable urease activity in the external media, indicating that the observed urea effect was the result of uptake of urea (**Figure 7A**). Even though urease activity was found in the extracts of crushed seeds (**Figure 7B**), its activity was orders of magnitude lower than in soil and presumably inadequate to produce any metabolic effect through a downstream product of urea metabolism. Whether this chemical effect of urea was direct or through a signaling cascade is an interesting aspect for further study.

In Arabidopsis seeds, Zonia et al. (1995) reported that the inhibition of urease activity delayed germination or caused failure to germinate. The suggestion by Qi et al. (2012) and Wan et al. (2016) that the inhibitory effect of urea on germination was due to ammonium toxicity from urease activity in the soil does not apply in our case for three reasons: Firstly, we show the inhibition of germination by nitrate as well as by urea (**Figure 2**). Secondly, the urea effect is seen even in the absence of external urease in our case (**Figure 7**), while the soil urease may be very high in their case. Thirdly, the very low level of urease in more than 2 g crushed seeds as compared to the high activity even in a few milligram of soil (control) shows that endogenous urease was inadequate to produce ammonia toxicity in our case (**Figure 7B**).

Interestingly, comparative quantitative assays for urease activity in contrasting genotypes revealed that slow germinating/respiring (long duration) genotypes had significantly lower urease activity (by 18.5%) as compared to the fast germinating (short duration) genotypes (*P* < 0.05, student’s *t*-test, **Figure 7C**), despite internal variation between individual genotypes. The presence of urea transporters (Wang et al., 2012), urease activity and internal urea metabolism in rice is known (Cao et al., 2010). But there is no literature to the best of our knowledge on the endogenous urease activity in seeds and the role of endogenous urease in the N response and/or NUE of rice or any other cereal crop. This could be due to the regional differences in the use of urea as fertilizer. Our preliminary findings on the differential responses of genotypes to urea and the 18.5% difference in urease activities between contrasting genotypes point to the need for further investigations regarding the metabolic role of urea and urease in N response and NUE. They could be particularly relevant to rice growing countries of South and East Asia, which are also the main markets for urea.

To understand the effect of N on events post-germination, we analyzed the early root and shoot growth using three contrasting pairs of genotypes germinated on agar media (**Figure 8**). While it revealed significant effects of N on both shoot and root length when all the N treatments and genotypes were taken together (*P* < 0.05, ANOVA), there was only significant inhibition of root length but not shoot length (*P* < 0.05, ANOVA) by any individual N source on any or all individual genotypes (**Figures 8B,C**). Urea not only had the most inhibitory effect on root length (**Figures 8A,B**), but also showed a dose–response relationship (**Figure 8D**), which was not found with shoot length (**Figure 8E**). Moreover, there was differential inhibition of root length by urea in contrasting groups of genotypes despite individual variations, indicating the need for further exploration of its potential for screening. The molecular mechanisms of these findings are also worthy of further exploration, since inhibition of cyanide insensitive oxygen consumption by alternative oxidase in rice was also shown to inhibit root growth (Sitaramam et al., 2008b).

The inhibitory effect of urea on root length in the soil was attributed to ammonia toxicity (Qi et al., 2012; Wan et al., 2016), but this may not apply to our findings in sterile agar media for the same reasons as discussed above in the context of urease experiments. In view of the strong phenotypic association between root growth parameters and NUE (Xu et al., 2012; Li et al., 2015; Xie et al., 2017), the inhibition of root growth by urea should be of serious concern in Asian countries where urea is the predominant form of N-fertilizer and broadcasting large quantities very few times is the main method of application.

## Conclusion

Our findings provide adequate evidence that the germination rate (t_½_), the influence of N on it (Δt_½_) and crop duration determine yield and NUE, at least in the Indica subspecies of rice. They can be further evaluated on a larger scale for their use as simple phenotyping tools to screen the rice germplasm for NUE. The fact that the 21 Indica genotypes used in this study represent diverse agroclimates, crop durations, and yields lends further credence to such a conclusion. The effect of N on O_2_ consumption points to the regulation of oxygen consumption as a possible mechanism for further investigation. The genotypic differences in urea uptake/utilization and urease activities remain a novel observation for further research in a hitherto unexplored area of research.

## Author Contributions

NS performed most of the experiments, data analysis, and wrote the first draft. VBS helped in early germination experiments. NG performed biplot analysis. SR performed qualitative urease tests. SK conducted the field experiments. VS suggested the exploration of the role of germination and respiration in NUE. RP provided statistical suggestions. NR helped in the planning, mentoring and supervision of the experiments, data interpretation and manuscript preparation.

## Conflict of Interest Statement

The authors declare that the research was conducted in the absence of any commercial or financial relationships that could be construed as a potential conflict of interest.

## Footnotes

1 http://statistics.amis-outlook.org/data/index.html#COMPARE

2 agricoop.nic.in

## Supplementary Material

FIGURES S1–21 Germination rates in media with N and without N.

TABLE S1 Correlation analysis ofthe ranking of 21 geno types by Kendall’s tau_b test. (a) Ranking based on time taken for 50% seeds to germinate (X_0_) and (b) ranking based on the effect of N on the time taken for 50% seeds to germinate (ΔX_0_). X_0_ values were obtained as described in the legend and the difference between the X_0_ values with and without N were calculated as ΔX_0_. Values in the parenthesis shows significance.

TABLE S2 Mean field data for grain yield and crop duration for 15 rice geno types grown without added N(N0)or with 100 Kg/ha urea N(N100) for 3 years over 6 seasons (2 seasons/year, Rabi and Kharif).
