# Supplementary material for: Phenotyping for Nitrogen Use Efficiency: Rice Genotypes Differ in N-Responsive Germination, Oxygen Consumption, Seed Urease Activities, Root Growth, Crop Duration, and Yield at Low N
Source: Front Plant Sci. 2018 Oct 1;9:1452. doi: 10.3389/fpls.2018.01452 (PMC6174359; doi:10.3389/fpls.2018.01452)
Supplement: FIGURES S1–21 — Germination rates in media with N and without N. [file Presentation_1.PPTX]

## Slide 1
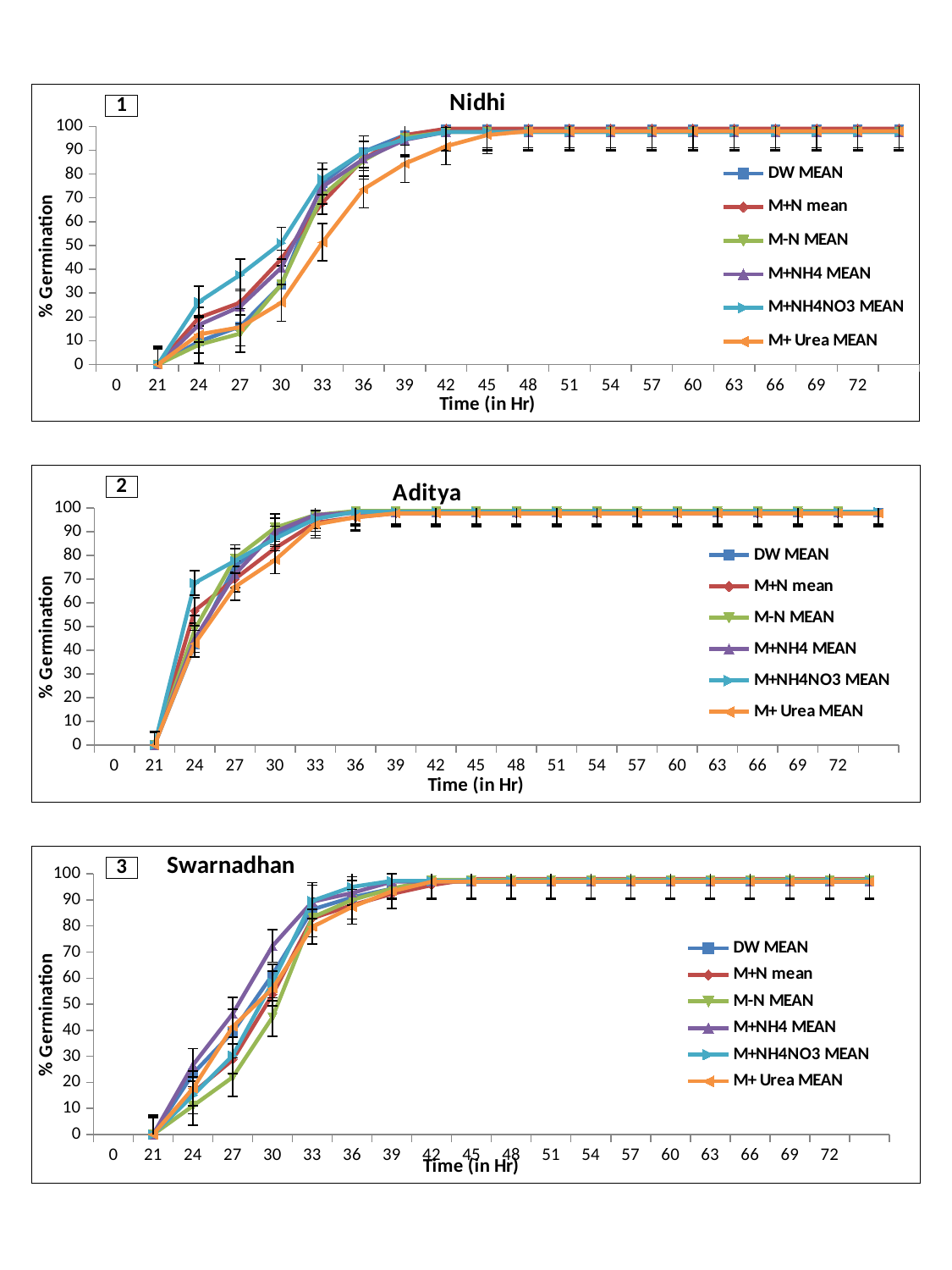

### Chart: Nidhi
| Category | DW MEAN | M+N mean | M-N MEAN | M+NH4 MEAN | M+NH4NO3 MEAN | M+ Urea MEAN |
|---|---|---|---|---|---|---|
| 0 | None | None | None | None | None | None |
| 21 | 0.0 | 0.0 | 0.0 | 0.0 | 0.0 | 0.0 |
| 24 | 9.666666666666677 | 19.666666666666668 | 8.333333333333336 | 16.666666666666668 | 26.33333333333324 | 12.666666666666694 |
| 27 | 16.0 | 26.0 | 13.0 | 24.33333333333324 | 37.66666666666647 | 15.666666666666694 |
| 30 | 33.333333333333336 | 44.333333333333336 | 33.66666666666647 | 40.66666666666647 | 51.0 | 26.0 |
| 33 | 76.33333333333326 | 68.0 | 71.0 | 74.66666666666667 | 78.0 | 51.333333333333336 |
| 36 | 89.33333333333326 | 86.33333333333326 | 85.66666666666667 | 86.33333333333326 | 89.33333333333326 | 73.66666666666667 |
| 39 | 96.33333333333326 | 96.33333333333326 | 95.33333333333326 | 94.33333333333326 | 94.66666666666667 | 84.33333333333326 |
| 42 | 98.66666666666667 | 99.0 | 97.66666666666667 | 97.66666666666667 | 97.66666666666667 | 91.66666666666667 |
| 45 | 98.66666666666667 | 99.0 | 97.66666666666667 | 97.66666666666667 | 97.66666666666667 | 96.33333333333326 |
| 48 | 98.66666666666667 | 99.0 | 97.66666666666667 | 97.66666666666667 | 97.66666666666667 | 98.0 |
| 51 | 98.66666666666667 | 99.0 | 97.66666666666667 | 97.66666666666667 | 97.66666666666667 | 98.0 |
| 54 | 98.66666666666667 | 99.0 | 97.66666666666667 | 97.66666666666667 | 97.66666666666667 | 98.0 |
| 57 | 98.66666666666667 | 99.0 | 97.66666666666667 | 97.66666666666667 | 97.66666666666667 | 98.0 |
| 60 | 98.66666666666667 | 99.0 | 97.66666666666667 | 97.66666666666667 | 97.66666666666667 | 98.0 |
| 63 | 98.66666666666667 | 99.0 | 97.66666666666667 | 97.66666666666667 | 97.66666666666667 | 98.0 |
| 66 | 98.66666666666667 | 99.0 | 97.66666666666667 | 97.66666666666667 | 97.66666666666667 | 98.0 |
| 69 | 98.66666666666667 | 99.0 | 97.66666666666667 | 97.66666666666667 | 97.66666666666667 | 98.0 |
| 72 | 98.66666666666667 | 99.0 | 97.66666666666667 | 97.66666666666667 | 97.66666666666667 | 98.0 |
### Chart: Aditya
| Category | DW MEAN | M+N mean | M-N MEAN | M+NH4 MEAN | M+NH4NO3 MEAN | M+ Urea MEAN |
|---|---|---|---|---|---|---|
| 0 | None | None | None | None | None | None |
| 21 | 0.0 | 0.0 | 0.0 | 0.0 | 0.0 | 0.0 |
| 24 | 42.333333333333336 | 56.66666666666647 | 48.66666666666647 | 44.66666666666647 | 68.33333333333326 | 42.66666666666647 |
| 27 | 75.33333333333326 | 70.0 | 78.66666666666667 | 72.0 | 77.66666666666667 | 66.66666666666667 |
| 30 | 88.33333333333326 | 83.0 | 91.66666666666667 | 90.0 | 87.0 | 78.0 |
| 33 | 96.33333333333326 | 93.66666666666667 | 97.0 | 97.0 | 95.33333333333326 | 93.0 |
| 36 | 98.0 | 96.0 | 98.66666666666667 | 98.0 | 98.33333333333326 | 96.0 |
| 39 | 98.0 | 97.66666666666667 | 98.66666666666667 | 98.0 | 98.33333333333326 | 97.66666666666667 |
| 42 | 98.0 | 97.66666666666667 | 98.66666666666667 | 98.0 | 98.33333333333326 | 97.66666666666667 |
| 45 | 98.0 | 97.66666666666667 | 98.66666666666667 | 98.0 | 98.33333333333326 | 97.66666666666667 |
| 48 | 98.0 | 97.66666666666667 | 98.66666666666667 | 98.0 | 98.33333333333326 | 97.66666666666667 |
| 51 | 98.0 | 97.66666666666667 | 98.66666666666667 | 98.0 | 98.33333333333326 | 97.66666666666667 |
| 54 | 98.0 | 97.66666666666667 | 98.66666666666667 | 98.0 | 98.33333333333326 | 97.66666666666667 |
| 57 | 98.0 | 97.66666666666667 | 98.66666666666667 | 98.0 | 98.33333333333326 | 97.66666666666667 |
| 60 | 98.0 | 97.66666666666667 | 98.66666666666667 | 98.0 | 98.33333333333326 | 97.66666666666667 |
| 63 | 98.0 | 97.66666666666667 | 98.66666666666667 | 98.0 | 98.33333333333326 | 97.66666666666667 |
| 66 | 98.0 | 97.66666666666667 | 98.66666666666667 | 98.0 | 98.33333333333326 | 97.66666666666667 |
| 69 | 98.0 | 97.66666666666667 | 98.66666666666667 | 98.0 | 98.33333333333326 | 97.66666666666667 |
| 72 | 98.0 | 97.66666666666667 | 98.66666666666667 | 98.0 | 98.33333333333326 | 97.66666666666667 |
### Chart: Swarnadhan
| Category | DW MEAN | M+N mean | M-N MEAN | M+NH4 MEAN | M+NH4NO3 MEAN | M+ Urea MEAN |
|---|---|---|---|---|---|---|
| 0 | None | None | None | None | None | None |
| 21 | 0.0 | 0.0 | 0.0 | 0.0 | 0.0 | 0.0 |
| 24 | 23.33333333333324 | 16.0 | 11.0 | 26.666666666666668 | 15.0 | 17.666666666666668 |
| 27 | 39.333333333333336 | 28.666666666666668 | 22.0 | 46.333333333333336 | 30.33333333333324 | 41.333333333333336 |
| 30 | 61.333333333333336 | 53.66666666666647 | 45.0 | 72.33333333333326 | 58.333333333333336 | 56.0 |
| 33 | 86.33333333333326 | 83.0 | 83.33333333333326 | 89.33333333333326 | 89.66666666666667 | 79.66666666666667 |
| 36 | 91.0 | 88.0 | 90.0 | 92.66666666666667 | 95.0 | 87.33333333333326 |
| 39 | 94.33333333333326 | 92.33333333333326 | 94.33333333333326 | 97.0 | 97.33333333333326 | 93.33333333333326 |
| 42 | 97.0 | 95.66666666666667 | 97.66666666666667 | 97.0 | 97.33333333333326 | 97.0 |
| 45 | 97.0 | 98.0 | 97.66666666666667 | 97.0 | 97.33333333333326 | 97.0 |
| 48 | 97.0 | 98.0 | 97.66666666666667 | 97.0 | 97.33333333333326 | 97.0 |
| 51 | 97.0 | 98.0 | 97.66666666666667 | 97.0 | 97.33333333333326 | 97.0 |
| 54 | 97.0 | 98.0 | 97.66666666666667 | 97.0 | 97.33333333333326 | 97.0 |
| 57 | 97.0 | 98.0 | 97.66666666666667 | 97.0 | 97.33333333333326 | 97.0 |
| 60 | 97.0 | 98.0 | 97.66666666666667 | 97.0 | 97.33333333333326 | 97.0 |
| 63 | 97.0 | 98.0 | 97.66666666666667 | 97.0 | 97.33333333333326 | 97.0 |
| 66 | 97.0 | 98.0 | 97.66666666666667 | 97.0 | 97.33333333333326 | 97.0 |
| 69 | 97.0 | 98.0 | 97.66666666666667 | 97.0 | 97.33333333333326 | 97.0 |
| 72 | 97.0 | 98.0 | 97.66666666666667 | 97.0 | 97.33333333333326 | 97.0 |

## Slide 2
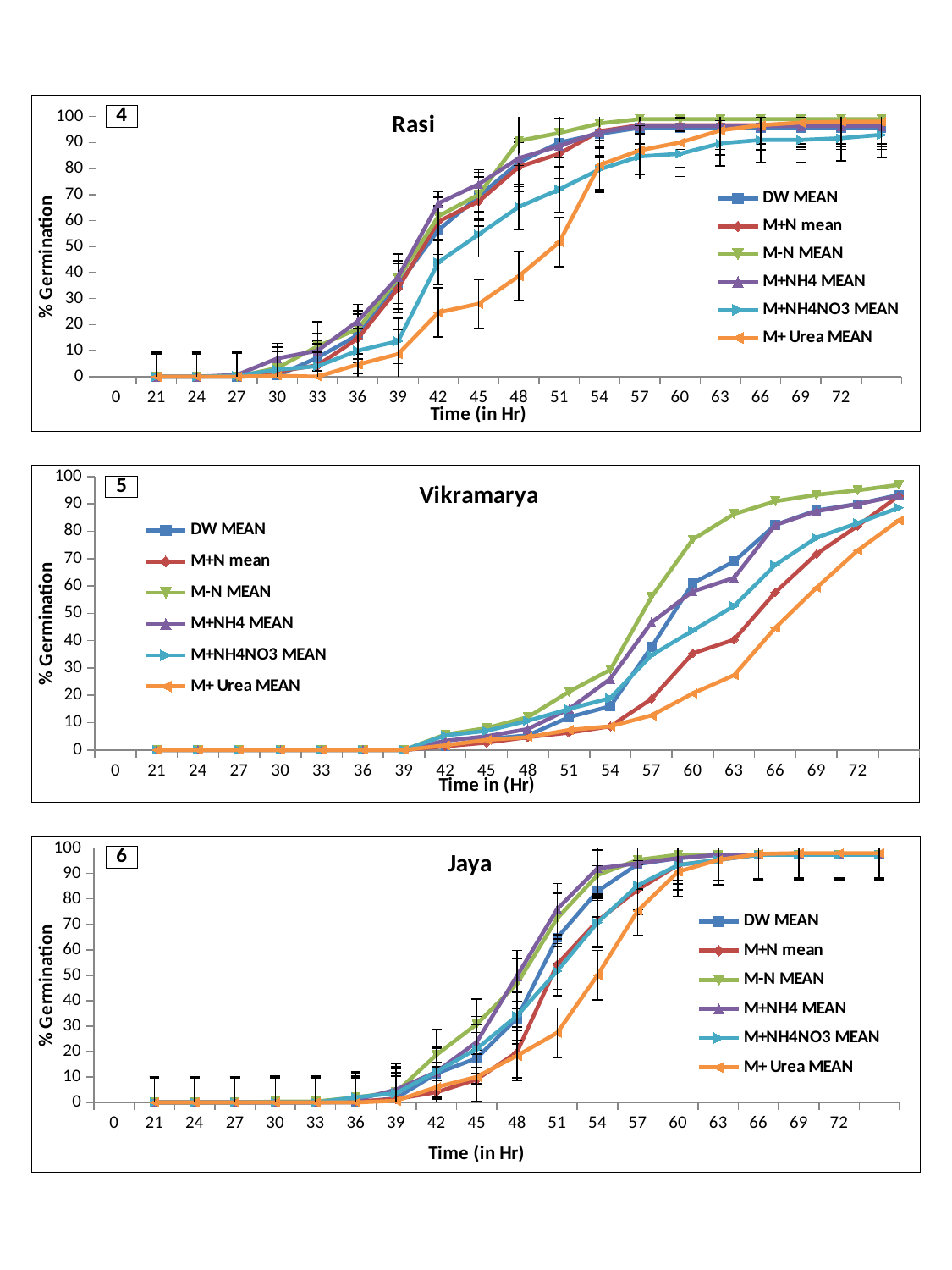

### Chart: Rasi
| Category | DW MEAN | M+N mean | M-N MEAN | M+NH4 MEAN | M+NH4NO3 MEAN | M+ Urea MEAN |
|---|---|---|---|---|---|---|
| 0 | None | None | None | None | None | None |
| 21 | 0.0 | 0.0 | 0.0 | 0.0 | 0.0 | 0.0 |
| 24 | 0.0 | 0.0 | 0.0 | 0.0 | 0.0 | 0.0 |
| 27 | 0.0 | 0.0 | 0.0 | 0.6666666666666666 | 0.3333333333333333 | 0.0 |
| 30 | 0.3333333333333333 | 2.0 | 3.3333333333333335 | 7.0 | 2.6666666666666665 | 0.3333333333333333 |
| 33 | 7.333333333333348 | 4.333333333333348 | 11.666666666666694 | 10.0 | 4.0 | 0.0 |
| 36 | 16.0 | 14.666666666666694 | 18.33333333333324 | 21.33333333333324 | 10.0 | 4.666666666666667 |
| 39 | 35.333333333333336 | 34.0 | 37.66666666666647 | 38.333333333333336 | 13.666666666666694 | 8.666666666666677 |
| 42 | 56.333333333333336 | 59.66666666666647 | 61.66666666666647 | 66.66666666666667 | 44.0 | 24.666666666666668 |
| 45 | 69.33333333333326 | 67.33333333333326 | 70.0 | 74.0 | 54.66666666666647 | 28.0 |
| 48 | 82.33333333333326 | 80.66666666666667 | 90.66666666666667 | 84.0 | 65.33333333333326 | 38.66666666666647 |
| 51 | 90.0 | 85.66666666666667 | 93.66666666666667 | 88.66666666666667 | 72.0 | 51.66666666666647 |
| 54 | 93.33333333333326 | 94.33333333333326 | 97.33333333333326 | 94.0 | 79.66666666666667 | 81.33333333333326 |
| 57 | 95.66666666666667 | 96.66666666666667 | 99.0 | 96.33333333333326 | 84.66666666666667 | 87.0 |
| 60 | 95.66666666666667 | 96.66666666666667 | 99.0 | 96.33333333333326 | 85.66666666666667 | 90.0 |
| 63 | 95.66666666666667 | 96.66666666666667 | 99.0 | 96.33333333333326 | 89.66666666666667 | 94.66666666666667 |
| 66 | 95.66666666666667 | 96.66666666666667 | 99.0 | 96.33333333333326 | 91.0 | 96.66666666666667 |
| 69 | 95.66666666666667 | 96.66666666666667 | 99.0 | 96.33333333333326 | 91.0 | 97.66666666666667 |
| 72 | 95.66666666666667 | 96.66666666666667 | 99.0 | 96.33333333333326 | 91.66666666666667 | 98.0 |
### Chart: Vikramarya
| Category | DW MEAN | M+N mean | M-N MEAN | M+NH4 MEAN | M+NH4NO3 MEAN | M+ Urea MEAN |
|---|---|---|---|---|---|---|
| 0 | None | None | None | None | None | None |
| 21 | 0.0 | 0.0 | 0.0 | 0.0 | 0.0 | 0.0 |
| 24 | 0.0 | 0.0 | 0.0 | 0.0 | 0.0 | 0.0 |
| 27 | 0.0 | 0.0 | 0.0 | 0.0 | 0.0 | 0.0 |
| 30 | 0.0 | 0.0 | 0.0 | 0.0 | 0.0 | 0.0 |
| 33 | 0.0 | 0.0 | 0.0 | 0.0 | 0.0 | 0.0 |
| 36 | 0.0 | 0.0 | 0.0 | 0.0 | 0.0 | 0.0 |
| 39 | 0.0 | 0.0 | 0.0 | 0.0 | 0.0 | 0.0 |
| 42 | 1.3333333333333333 | 1.3333333333333333 | 5.666666666666667 | 3.3333333333333335 | 5.333333333333348 | 1.6666666666666667 |
| 45 | 4.0 | 2.6666666666666665 | 8.0 | 5.0 | 7.0 | 3.6666666666666665 |
| 48 | 5.333333333333348 | 4.666666666666667 | 12.0 | 7.666666666666667 | 10.666666666666694 | 4.666666666666667 |
| 51 | 12.0 | 6.333333333333348 | 21.33333333333324 | 15.0 | 15.0 | 7.333333333333348 |
| 54 | 16.0 | 8.666666666666677 | 29.33333333333324 | 26.0 | 19.0 | 8.666666666666677 |
| 57 | 37.66666666666647 | 18.666666666666668 | 56.0 | 46.66666666666647 | 34.66666666666647 | 12.666666666666694 |
| 60 | 61.0 | 35.333333333333336 | 77.0 | 58.0 | 43.66666666666647 | 20.666666666666668 |
| 63 | 69.0 | 40.333333333333336 | 86.33333333333326 | 63.0 | 52.66666666666647 | 27.33333333333324 |
| 66 | 82.33333333333326 | 57.66666666666647 | 91.0 | 82.33333333333326 | 67.66666666666667 | 44.66666666666647 |
| 69 | 87.66666666666667 | 71.66666666666667 | 93.33333333333326 | 87.33333333333326 | 77.66666666666667 | 59.333333333333336 |
| 72 | 90.0 | 82.0 | 95.0 | 90.0 | 83.0 | 73.0 |
### Chart: Jaya
| Category | DW MEAN | M+N mean | M-N MEAN | M+NH4 MEAN | M+NH4NO3 MEAN | M+ Urea MEAN |
|---|---|---|---|---|---|---|
| 0 | None | None | None | None | None | None |
| 21 | 0.0 | 0.0 | 0.0 | 0.0 | 0.0 | 0.0 |
| 24 | 0.0 | 0.0 | 0.0 | 0.0 | 0.0 | 0.0 |
| 27 | 0.0 | 0.0 | 0.0 | 0.0 | 0.0 | 0.0 |
| 30 | 0.0 | 0.0 | 0.3333333333333333 | 0.0 | 0.0 | 0.0 |
| 33 | 0.0 | 0.0 | 0.3333333333333333 | 0.0 | 0.0 | 0.0 |
| 36 | 0.0 | 0.3333333333333333 | 2.0 | 1.0 | 2.0 | 0.0 |
| 39 | 1.6666666666666667 | 1.3333333333333333 | 4.0 | 5.0 | 3.6666666666666665 | 0.6666666666666666 |
| 42 | 11.333333333333334 | 4.0 | 18.666666666666668 | 12.0 | 12.0 | 6.0 |
| 45 | 17.33333333333324 | 9.0 | 30.666666666666668 | 23.666666666666668 | 21.0 | 10.0 |
| 48 | 33.0 | 19.666666666666668 | 46.66666666666647 | 49.66666666666647 | 34.0 | 18.33333333333324 |
| 51 | 64.66666666666667 | 54.333333333333336 | 72.33333333333326 | 76.0 | 51.66666666666647 | 27.33333333333324 |
| 54 | 83.0 | 71.33333333333326 | 89.33333333333326 | 92.0 | 70.66666666666667 | 50.0 |
| 57 | 93.66666666666667 | 83.66666666666667 | 95.33333333333326 | 94.0 | 85.33333333333326 | 75.33333333333326 |
| 60 | 96.0 | 93.33333333333326 | 97.33333333333326 | 96.0 | 93.33333333333326 | 90.66666666666667 |
| 63 | 97.33333333333326 | 95.33333333333326 | 97.33333333333326 | 97.33333333333326 | 95.33333333333326 | 95.33333333333326 |
| 66 | 97.33333333333326 | 97.33333333333326 | 97.33333333333326 | 97.33333333333326 | 97.33333333333326 | 97.66666666666667 |
| 69 | 97.33333333333326 | 97.33333333333326 | 97.33333333333326 | 97.33333333333326 | 97.33333333333326 | 98.0 |
| 72 | 97.33333333333326 | 97.33333333333326 | 97.33333333333326 | 97.33333333333326 | 97.33333333333326 | 98.0 |

## Slide 3
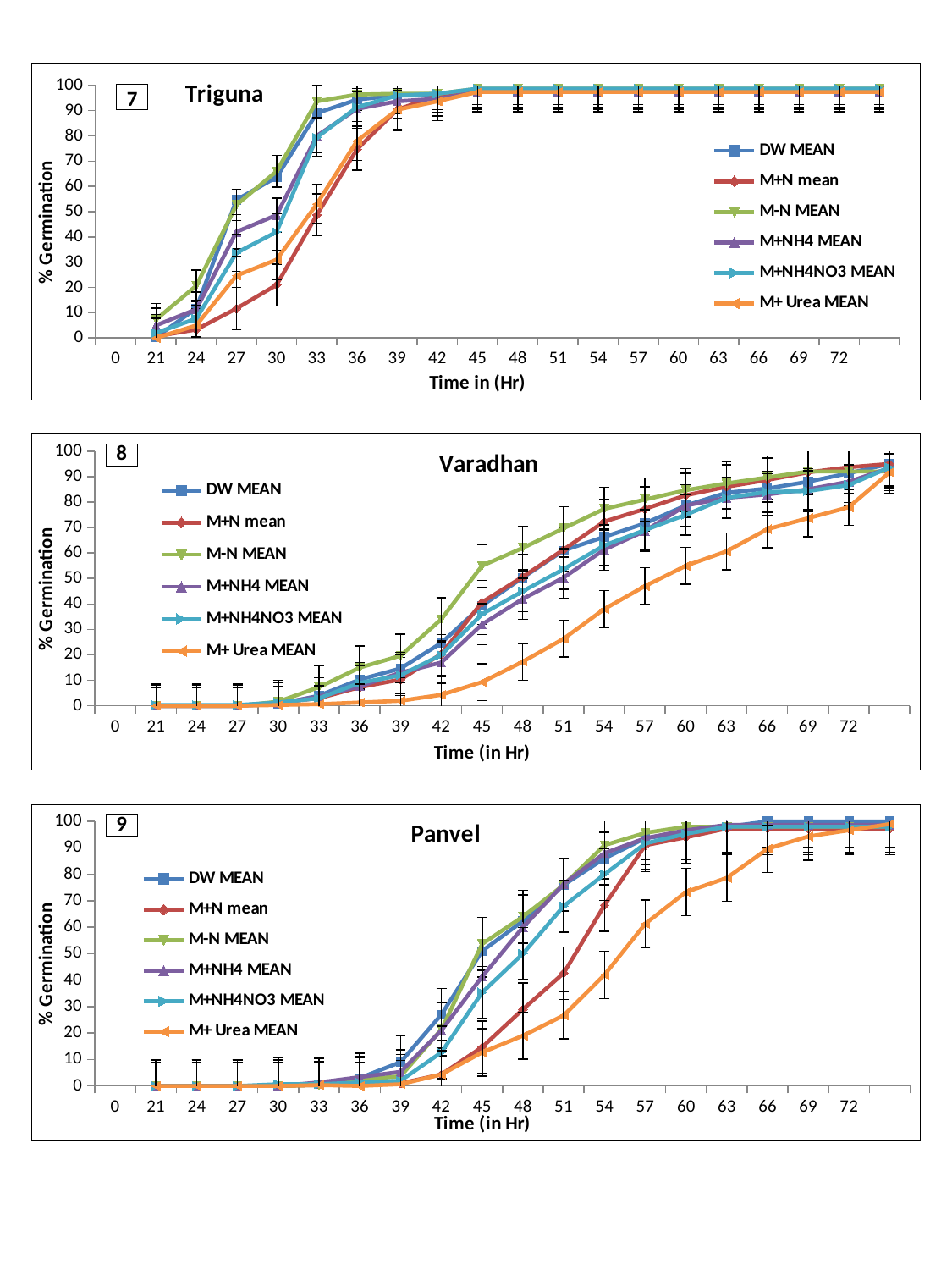

### Chart: Triguna
| Category | DW MEAN | M+N mean | M-N MEAN | M+NH4 MEAN | M+NH4NO3 MEAN | M+ Urea MEAN |
|---|---|---|---|---|---|---|
| 0 | None | None | None | None | None | None |
| 21 | 0.3333333333333333 | 0.6666666666666666 | 7.333333333333348 | 5.0 | 2.0 | 0.0 |
| 24 | 11.666666666666694 | 3.3333333333333335 | 20.666666666666668 | 11.333333333333334 | 7.666666666666667 | 5.0 |
| 27 | 54.66666666666647 | 11.666666666666694 | 52.66666666666647 | 42.0 | 33.66666666666647 | 24.666666666666668 |
| 30 | 63.66666666666647 | 21.0 | 66.0 | 48.66666666666647 | 42.0 | 31.0 |
| 33 | 89.0 | 48.66666666666647 | 93.66666666666667 | 80.0 | 79.33333333333326 | 53.0 |
| 36 | 94.33333333333326 | 74.66666666666667 | 96.33333333333326 | 90.66666666666667 | 91.33333333333326 | 78.0 |
| 39 | 96.0 | 90.33333333333326 | 96.66666666666667 | 93.66666666666667 | 96.0 | 90.33333333333326 |
| 42 | 96.0 | 96.0 | 96.66666666666667 | 94.66666666666667 | 96.66666666666667 | 93.66666666666667 |
| 45 | 97.66666666666667 | 98.0 | 98.66666666666667 | 97.33333333333326 | 98.66666666666667 | 97.33333333333326 |
| 48 | 97.66666666666667 | 98.0 | 98.66666666666667 | 97.33333333333326 | 98.66666666666667 | 97.33333333333326 |
| 51 | 97.66666666666667 | 98.0 | 98.66666666666667 | 97.33333333333326 | 98.66666666666667 | 97.33333333333326 |
| 54 | 97.66666666666667 | 98.0 | 98.66666666666667 | 97.33333333333326 | 98.66666666666667 | 97.33333333333326 |
| 57 | 97.66666666666667 | 98.0 | 98.66666666666667 | 97.33333333333326 | 98.66666666666667 | 97.33333333333326 |
| 60 | 97.66666666666667 | 98.0 | 98.66666666666667 | 97.33333333333326 | 98.66666666666667 | 97.33333333333326 |
| 63 | 97.66666666666667 | 98.0 | 98.66666666666667 | 97.33333333333326 | 98.66666666666667 | 97.33333333333326 |
| 66 | 97.66666666666667 | 98.0 | 98.66666666666667 | 97.33333333333326 | 98.66666666666667 | 97.33333333333326 |
| 69 | 97.66666666666667 | 98.0 | 98.66666666666667 | 97.33333333333326 | 98.66666666666667 | 97.33333333333326 |
| 72 | 97.66666666666667 | 98.0 | 98.66666666666667 | 97.33333333333326 | 98.66666666666667 | 97.33333333333326 |7
### Chart: Varadhan
| Category | DW MEAN | M+N mean | M-N MEAN | M+NH4 MEAN | M+NH4NO3 MEAN | M+ Urea MEAN |
|---|---|---|---|---|---|---|
| 0 | None | None | None | None | None | None |
| 21 | 0.0 | 0.0 | 0.0 | 0.0 | 0.3333333333333333 | 0.0 |
| 24 | 0.0 | 0.0 | 0.0 | 0.0 | 0.3333333333333333 | 0.0 |
| 27 | 0.0 | 0.0 | 0.0 | 0.0 | 0.3333333333333333 | 0.0 |
| 30 | 0.6666666666666666 | 1.3333333333333333 | 1.6666666666666667 | 1.0 | 1.3333333333333333 | 0.3333333333333333 |
| 33 | 4.0 | 3.0 | 7.333333333333348 | 3.6666666666666665 | 3.0 | 0.6666666666666666 |
| 36 | 10.333333333333334 | 7.333333333333348 | 15.0 | 7.666666666666667 | 9.0 | 1.3333333333333333 |
| 39 | 14.666666666666694 | 10.333333333333334 | 19.666666666666668 | 13.0 | 12.0 | 2.0 |
| 42 | 24.666666666666668 | 20.33333333333324 | 34.0 | 17.0 | 20.0 | 4.333333333333348 |
| 45 | 39.333333333333336 | 40.66666666666647 | 55.0 | 32.0 | 36.0 | 9.333333333333336 |
| 48 | 50.333333333333336 | 50.66666666666647 | 62.0 | 42.0 | 45.0 | 17.33333333333324 |
| 51 | 61.0 | 61.333333333333336 | 69.66666666666667 | 50.333333333333336 | 53.66666666666647 | 26.33333333333324 |
| 54 | 66.33333333333326 | 72.33333333333326 | 77.33333333333326 | 61.333333333333336 | 63.0 | 38.0 |
| 57 | 71.66666666666667 | 77.33333333333326 | 81.0 | 68.66666666666667 | 69.0 | 47.0 |
| 60 | 78.66666666666667 | 82.66666666666667 | 84.66666666666667 | 78.66666666666667 | 75.0 | 55.0 |
| 63 | 83.66666666666667 | 86.0 | 87.33333333333326 | 81.66666666666667 | 81.66666666666667 | 60.66666666666647 |
| 66 | 85.33333333333326 | 88.66666666666667 | 89.66666666666667 | 83.0 | 84.0 | 69.33333333333326 |
| 69 | 88.0 | 91.66666666666667 | 92.0 | 85.0 | 84.33333333333326 | 73.66666666666667 |
| 72 | 91.33333333333326 | 93.66666666666667 | 92.0 | 88.0 | 86.66666666666667 | 78.0 |
### Chart: Panvel
| Category | DW MEAN | M+N mean | M-N MEAN | M+NH4 MEAN | M+NH4NO3 MEAN | M+ Urea MEAN |
|---|---|---|---|---|---|---|
| 0 | None | None | None | None | None | None |
| 21 | 0.0 | 0.0 | 0.0 | 0.0 | 0.0 | 0.0 |
| 24 | 0.0 | 0.0 | 0.0 | 0.0 | 0.0 | 0.0 |
| 27 | 0.0 | 0.0 | 0.0 | 0.0 | 0.0 | 0.0 |
| 30 | 0.0 | 0.0 | 0.0 | 0.0 | 0.6666666666666666 | 0.0 |
| 33 | 0.6666666666666666 | 0.3333333333333333 | 0.3333333333333333 | 1.3333333333333333 | 0.6666666666666666 | 0.3333333333333333 |
| 36 | 3.0 | 0.6666666666666666 | 2.3333333333333335 | 3.3333333333333335 | 1.3333333333333333 | 0.0 |
| 39 | 9.0 | 1.0 | 3.6666666666666665 | 5.333333333333348 | 2.0 | 0.6666666666666666 |
| 42 | 27.0 | 4.333333333333348 | 21.33333333333324 | 21.0 | 12.666666666666694 | 4.333333333333348 |
| 45 | 51.0 | 14.666666666666694 | 53.66666666666647 | 41.333333333333336 | 35.333333333333336 | 12.666666666666694 |
| 48 | 62.333333333333336 | 29.0 | 64.0 | 60.0 | 50.0 | 19.0 |
| 51 | 76.0 | 42.66666666666647 | 76.0 | 76.33333333333326 | 68.0 | 26.666666666666668 |
| 54 | 86.0 | 68.33333333333326 | 91.0 | 88.0 | 80.0 | 42.0 |
| 57 | 93.66666666666667 | 91.0 | 95.66666666666667 | 93.66666666666667 | 91.66666666666667 | 61.333333333333336 |
| 60 | 95.66666666666667 | 94.0 | 98.0 | 96.66666666666667 | 95.33333333333326 | 73.33333333333326 |
| 63 | 98.0 | 97.33333333333326 | 98.0 | 98.66666666666667 | 98.0 | 78.66666666666667 |
| 66 | 100.0 | 97.33333333333326 | 98.0 | 98.66666666666667 | 98.0 | 89.66666666666667 |
| 69 | 100.0 | 97.33333333333326 | 98.0 | 98.66666666666667 | 98.0 | 94.33333333333326 |
| 72 | 100.0 | 97.33333333333326 | 98.0 | 98.66666666666667 | 98.0 | 96.66666666666667 |

## Slide 4
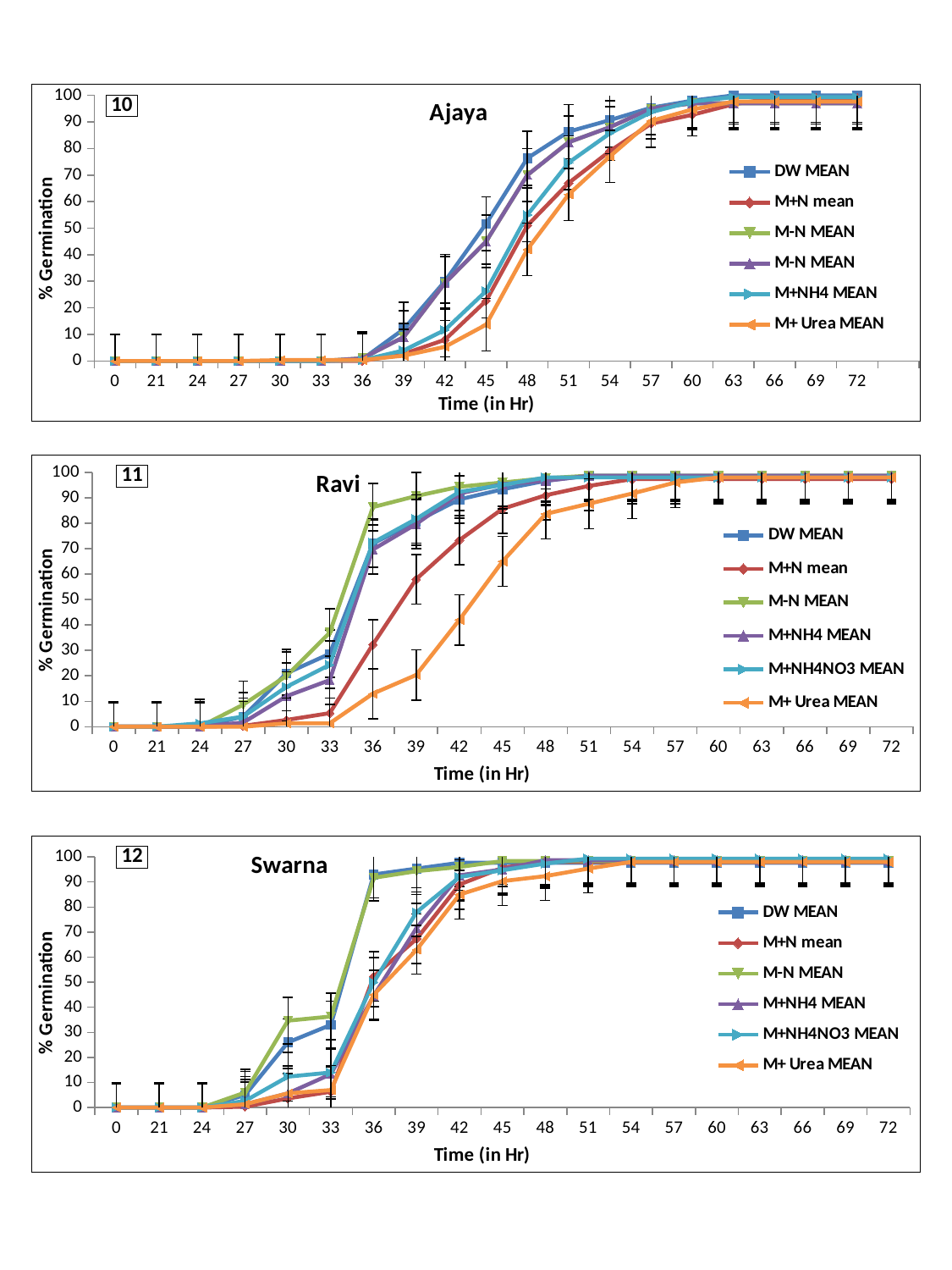

### Chart: Ajaya
| Category | DW MEAN | M+N mean | M-N MEAN | M-N MEAN | M+NH4 MEAN | M+ Urea MEAN |
|---|---|---|---|---|---|---|
| 0 | 0.0 | 0.0 | 0.0 | 0.0 | 0.0 | 0.0 |
| 21 | 0.0 | 0.0 | 0.0 | 0.0 | 0.0 | 0.0 |
| 24 | 0.0 | 0.0 | 0.0 | 0.0 | 0.0 | 0.0 |
| 27 | 0.0 | 0.0 | 0.0 | 0.0 | 0.0 | 0.0 |
| 30 | 0.0 | 0.0 | 0.0 | 0.0 | 0.0 | 0.3333333333333333 |
| 33 | 0.0 | 0.0 | 0.0 | 0.0 | 0.0 | 0.3333333333333333 |
| 36 | 0.6666666666666666 | 0.0 | 1.0 | 1.0 | 0.3333333333333333 | 0.3333333333333333 |
| 39 | 12.0 | 2.6666666666666665 | 9.0 | 9.0 | 4.0 | 2.0 |
| 42 | 30.0 | 8.0 | 29.33333333333324 | 29.33333333333324 | 11.666666666666694 | 5.333333333333348 |
| 45 | 51.66666666666647 | 22.666666666666668 | 45.0 | 45.0 | 26.33333333333324 | 13.666666666666694 |
| 48 | 76.33333333333326 | 51.0 | 70.0 | 70.0 | 55.0 | 42.0 |
| 51 | 86.33333333333326 | 67.0 | 82.33333333333326 | 82.33333333333326 | 74.66666666666667 | 62.66666666666647 |
| 54 | 90.66666666666667 | 79.0 | 88.0 | 88.0 | 85.66666666666667 | 77.0 |
| 57 | 95.33333333333326 | 89.33333333333326 | 95.0 | 95.0 | 93.66666666666667 | 90.33333333333326 |
| 60 | 98.0 | 92.66666666666667 | 97.0 | 97.0 | 97.66666666666667 | 94.66666666666667 |
| 63 | 100.0 | 96.66666666666667 | 97.0 | 97.0 | 99.33333333333326 | 97.66666666666667 |
| 66 | 100.0 | 99.0 | 97.0 | 97.0 | 99.33333333333326 | 97.66666666666667 |
| 69 | 100.0 | 99.0 | 97.0 | 97.0 | 99.33333333333326 | 97.66666666666667 |
| 72 | 100.0 | 99.0 | 97.0 | 97.0 | 99.33333333333326 | 97.66666666666667 |
### Chart: Ravi
| Category | DW MEAN | M+N mean | M-N MEAN | M+NH4 MEAN | M+NH4NO3 MEAN | M+ Urea MEAN |
|---|---|---|---|---|---|---|
| 0 | 0.0 | 0.0 | 0.0 | 0.0 | 0.0 | 0.0 |
| 21 | 0.0 | 0.0 | 0.0 | 0.0 | 0.0 | 0.0 |
| 24 | 0.0 | 0.0 | 0.0 | 0.0 | 1.3333333333333333 | 0.0 |
| 27 | 4.0 | 0.3333333333333333 | 8.666666666666677 | 1.6666666666666667 | 4.0 | 0.0 |
| 30 | 21.0 | 2.6666666666666665 | 20.0 | 12.0 | 15.666666666666694 | 1.3333333333333333 |
| 33 | 28.666666666666668 | 5.333333333333348 | 37.0 | 18.33333333333324 | 24.33333333333324 | 1.3333333333333333 |
| 36 | 72.0 | 32.333333333333336 | 86.33333333333326 | 69.66666666666667 | 72.33333333333326 | 13.0 |
| 39 | 80.66666666666667 | 58.0 | 90.66666666666667 | 79.66666666666667 | 81.66666666666667 | 20.33333333333324 |
| 42 | 89.33333333333326 | 73.33333333333326 | 94.33333333333326 | 91.66666666666667 | 92.33333333333326 | 42.0 |
| 45 | 93.33333333333326 | 85.66666666666667 | 96.0 | 95.33333333333326 | 95.0 | 65.0 |
| 48 | 96.66666666666667 | 91.0 | 97.66666666666667 | 96.66666666666667 | 98.0 | 83.66666666666667 |
| 51 | 98.66666666666667 | 94.66666666666667 | 98.66666666666667 | 98.66666666666667 | 98.0 | 87.66666666666667 |
| 54 | 98.66666666666667 | 97.33333333333326 | 98.66666666666667 | 98.66666666666667 | 98.0 | 91.66666666666667 |
| 57 | 98.66666666666667 | 97.33333333333326 | 98.66666666666667 | 98.66666666666667 | 98.0 | 96.0 |
| 60 | 98.66666666666667 | 97.33333333333326 | 98.66666666666667 | 98.66666666666667 | 98.0 | 98.0 |
| 63 | 98.66666666666667 | 97.33333333333326 | 98.66666666666667 | 98.66666666666667 | 98.0 | 98.0 |
| 66 | 98.66666666666667 | 97.33333333333326 | 98.66666666666667 | 98.66666666666667 | 98.0 | 98.0 |
| 69 | 98.66666666666667 | 97.33333333333326 | 98.66666666666667 | 98.66666666666667 | 98.0 | 98.0 |
| 72 | 98.66666666666667 | 97.33333333333326 | 98.66666666666667 | 98.66666666666667 | 98.0 | 98.0 |
### Chart: Swarna
| Category | DW MEAN | M+N mean | M-N MEAN | M+NH4 MEAN | M+NH4NO3 MEAN | M+ Urea MEAN |
|---|---|---|---|---|---|---|
| 0 | 0.0 | 0.0 | 0.0 | 0.0 | 0.0 | 0.0 |
| 21 | 0.0 | 0.0 | 0.0 | 0.0 | 0.0 | 0.0 |
| 24 | 0.0 | 0.0 | 0.0 | 0.0 | 0.0 | 0.0 |
| 27 | 5.0 | 0.3333333333333333 | 6.0 | 1.3333333333333333 | 2.6666666666666665 | 1.3333333333333333 |
| 30 | 26.0 | 3.6666666666666665 | 34.66666666666647 | 5.666666666666667 | 12.333333333333334 | 5.666666666666667 |
| 33 | 33.0 | 6.333333333333348 | 36.333333333333336 | 13.333333333333334 | 14.0 | 7.0 |
| 36 | 93.0 | 52.333333333333336 | 91.66666666666667 | 44.66666666666647 | 50.0 | 45.0 |
| 39 | 95.33333333333326 | 67.33333333333326 | 94.33333333333326 | 71.66666666666667 | 78.0 | 63.0 |
| 42 | 97.66666666666667 | 89.0 | 96.0 | 92.66666666666667 | 92.0 | 85.0 |
| 45 | 97.66666666666667 | 95.66666666666667 | 98.33333333333326 | 95.0 | 94.66666666666667 | 90.33333333333326 |
| 48 | 97.66666666666667 | 98.0 | 98.33333333333326 | 98.66666666666667 | 97.33333333333326 | 92.33333333333326 |
| 51 | 97.66666666666667 | 98.0 | 98.33333333333326 | 98.66666666666667 | 99.33333333333326 | 95.33333333333326 |
| 54 | 97.66666666666667 | 98.0 | 98.33333333333326 | 98.66666666666667 | 99.33333333333326 | 98.0 |
| 57 | 97.66666666666667 | 98.0 | 98.33333333333326 | 98.66666666666667 | 99.33333333333326 | 98.0 |
| 60 | 97.66666666666667 | 98.0 | 98.33333333333326 | 98.66666666666667 | 99.33333333333326 | 98.0 |
| 63 | 97.66666666666667 | 98.0 | 98.33333333333326 | 98.66666666666667 | 99.33333333333326 | 98.0 |
| 66 | 97.66666666666667 | 98.0 | 98.33333333333326 | 98.66666666666667 | 99.33333333333326 | 98.0 |
| 69 | 97.66666666666667 | 98.0 | 98.33333333333326 | 98.66666666666667 | 99.33333333333326 | 98.0 |
| 72 | 97.66666666666667 | 98.0 | 98.33333333333326 | 98.66666666666667 | 99.33333333333326 | 98.0 |

## Slide 5
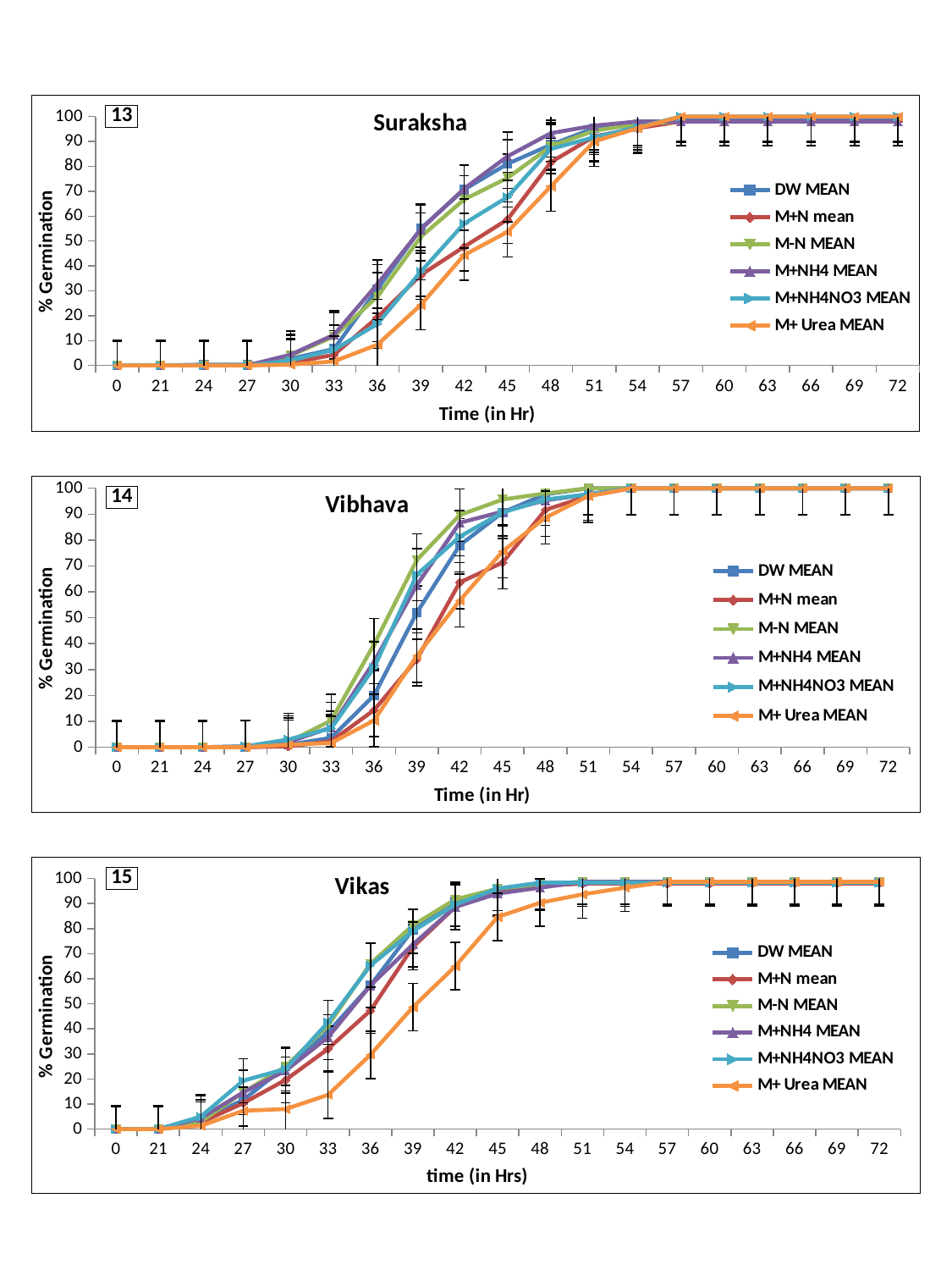

### Chart: Suraksha
| Category | DW MEAN | M+N mean | M-N MEAN | M+NH4 MEAN | M+NH4NO3 MEAN | M+ Urea MEAN |
|---|---|---|---|---|---|---|
| 0 | 0.0 | 0.0 | 0.0 | 0.0 | 0.0 | 0.0 |
| 21 | 0.0 | 0.0 | 0.0 | 0.0 | 0.0 | 0.0 |
| 24 | 0.3333333333333333 | 0.0 | 0.0 | 0.0 | 0.0 | 0.0 |
| 27 | 0.3333333333333333 | 0.0 | 0.0 | 0.0 | 0.0 | 0.0 |
| 30 | 2.6666666666666665 | 1.0 | 4.0 | 4.333333333333348 | 2.0 | 0.3333333333333333 |
| 33 | 6.666666666666667 | 4.333333333333348 | 11.666666666666694 | 12.333333333333334 | 6.333333333333348 | 1.6666666666666667 |
| 36 | 30.666666666666668 | 19.33333333333324 | 27.666666666666668 | 32.66666666666647 | 16.666666666666668 | 8.333333333333336 |
| 39 | 55.0 | 36.333333333333336 | 51.66666666666647 | 54.66666666666647 | 37.66666666666647 | 24.33333333333324 |
| 42 | 70.66666666666667 | 47.66666666666647 | 66.66666666666667 | 71.0 | 57.0 | 44.333333333333336 |
| 45 | 81.0 | 58.66666666666647 | 75.33333333333326 | 84.0 | 67.66666666666667 | 53.66666666666647 |
| 48 | 88.66666666666667 | 81.66666666666667 | 88.0 | 93.33333333333326 | 87.0 | 72.0 |
| 51 | 95.33333333333326 | 92.0 | 94.33333333333326 | 96.33333333333326 | 91.66666666666667 | 90.0 |
| 54 | 96.33333333333326 | 95.33333333333326 | 97.0 | 98.0 | 95.66666666666667 | 95.33333333333326 |
| 57 | 99.33333333333326 | 98.0 | 98.0 | 98.0 | 100.0 | 100.0 |
| 60 | 99.33333333333326 | 98.0 | 98.0 | 98.0 | 100.0 | 100.0 |
| 63 | 99.33333333333326 | 98.0 | 98.0 | 98.0 | 100.0 | 100.0 |
| 66 | 99.33333333333326 | 98.0 | 98.0 | 98.0 | 100.0 | 100.0 |
| 69 | 99.33333333333326 | 98.0 | 98.0 | 98.0 | 100.0 | 100.0 |
| 72 | 99.33333333333326 | 98.0 | 98.0 | 98.0 | 100.0 | 100.0 |
### Chart: Vibhava
| Category | DW MEAN | M+N mean | M-N MEAN | M+NH4 MEAN | M+NH4NO3 MEAN | M+ Urea MEAN |
|---|---|---|---|---|---|---|
| 0 | 0.0 | 0.0 | 0.0 | 0.0 | 0.0 | 0.0 |
| 21 | 0.0 | 0.0 | 0.0 | 0.0 | 0.0 | 0.0 |
| 24 | 0.0 | 0.0 | 0.0 | 0.0 | 0.0 | 0.0 |
| 27 | 0.0 | 0.0 | 0.0 | 0.3333333333333333 | 0.3333333333333333 | 0.0 |
| 30 | 1.0 | 0.3333333333333333 | 2.0 | 2.0 | 3.0 | 1.0 |
| 33 | 3.6666666666666665 | 2.3333333333333335 | 10.333333333333334 | 7.666666666666667 | 7.333333333333348 | 1.6666666666666667 |
| 36 | 20.0 | 14.333333333333334 | 39.66666666666647 | 33.0 | 30.666666666666668 | 10.333333333333334 |
| 39 | 52.0 | 34.0 | 72.33333333333326 | 62.66666666666647 | 66.66666666666667 | 35.333333333333336 |
| 42 | 78.0 | 63.66666666666647 | 89.66666666666667 | 86.66666666666667 | 81.33333333333326 | 56.66666666666647 |
| 45 | 90.66666666666667 | 71.33333333333326 | 95.66666666666667 | 91.0 | 90.66666666666667 | 75.66666666666667 |
| 48 | 97.66666666666667 | 91.66666666666667 | 98.0 | 95.33333333333326 | 95.66666666666667 | 88.66666666666667 |
| 51 | 100.0 | 97.0 | 100.0 | 97.66666666666667 | 97.66666666666667 | 97.0 |
| 54 | 100.0 | 100.0 | 100.0 | 100.0 | 100.0 | 100.0 |
| 57 | 100.0 | 100.0 | 100.0 | 100.0 | 100.0 | 100.0 |
| 60 | 100.0 | 100.0 | 100.0 | 100.0 | 100.0 | 100.0 |
| 63 | 100.0 | 100.0 | 100.0 | 100.0 | 100.0 | 100.0 |
| 66 | 100.0 | 100.0 | 100.0 | 100.0 | 100.0 | 100.0 |
| 69 | 100.0 | 100.0 | 100.0 | 100.0 | 100.0 | 100.0 |
| 72 | 100.0 | 100.0 | 100.0 | 100.0 | 100.0 | 100.0 |
### Chart: Vikas
| Category | DW MEAN | M+N mean | M-N MEAN | M+NH4 MEAN | M+NH4NO3 MEAN | M+ Urea MEAN |
|---|---|---|---|---|---|---|
| 0 | 0.0 | 0.0 | 0.0 | 0.0 | 0.0 | 0.0 |
| 21 | 0.0 | 0.0 | 0.0 | 0.0 | 0.0 | 0.0 |
| 24 | 3.3333333333333335 | 2.6666666666666665 | 3.0 | 4.333333333333348 | 5.0 | 1.3333333333333333 |
| 27 | 11.666666666666694 | 10.333333333333334 | 14.666666666666694 | 14.666666666666694 | 19.33333333333324 | 7.333333333333348 |
| 30 | 25.0 | 19.666666666666668 | 25.0 | 23.33333333333324 | 24.0 | 8.0 |
| 33 | 38.66666666666647 | 32.0 | 41.333333333333336 | 36.66666666666647 | 42.66666666666647 | 13.666666666666694 |
| 36 | 57.333333333333336 | 47.333333333333336 | 66.0 | 57.333333333333336 | 65.33333333333326 | 29.666666666666668 |
| 39 | 79.66666666666667 | 72.66666666666667 | 81.33333333333326 | 73.66666666666667 | 79.0 | 48.66666666666647 |
| 42 | 91.0 | 88.66666666666667 | 91.66666666666667 | 88.66666666666667 | 89.66666666666667 | 65.0 |
| 45 | 95.0 | 94.66666666666667 | 96.0 | 94.0 | 96.0 | 84.66666666666667 |
| 48 | 97.66666666666667 | 97.0 | 97.66666666666667 | 96.33333333333326 | 98.33333333333326 | 90.33333333333326 |
| 51 | 98.66666666666667 | 98.0 | 98.66666666666667 | 98.66666666666667 | 98.33333333333326 | 93.66666666666667 |
| 54 | 98.66666666666667 | 98.0 | 98.66666666666667 | 98.66666666666667 | 98.33333333333326 | 96.33333333333326 |
| 57 | 98.66666666666667 | 98.0 | 98.66666666666667 | 98.66666666666667 | 98.33333333333326 | 98.66666666666667 |
| 60 | 98.66666666666667 | 98.0 | 98.66666666666667 | 98.66666666666667 | 98.33333333333326 | 98.66666666666667 |
| 63 | 98.66666666666667 | 98.0 | 98.66666666666667 | 98.66666666666667 | 98.33333333333326 | 98.66666666666667 |
| 66 | 98.66666666666667 | 98.0 | 98.66666666666667 | 98.66666666666667 | 98.33333333333326 | 98.66666666666667 |
| 69 | 98.66666666666667 | 98.0 | 98.66666666666667 | 98.66666666666667 | 98.33333333333326 | 98.66666666666667 |
| 72 | 98.66666666666667 | 98.0 | 98.66666666666667 | 98.66666666666667 | 98.33333333333326 | 98.66666666666667 |

## Slide 6
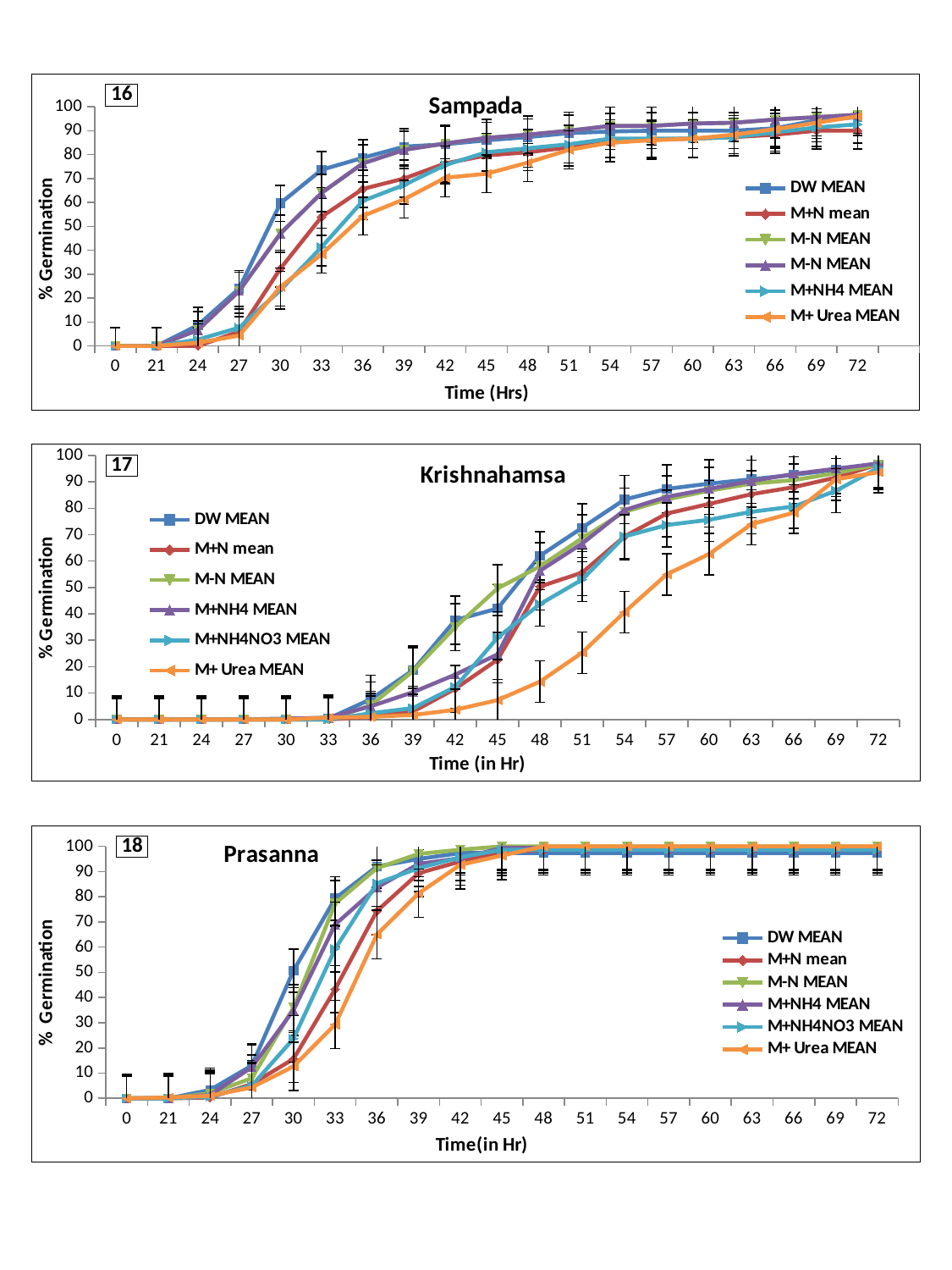

### Chart: Sampada
| Category | DW MEAN | M+N mean | M-N MEAN | M-N MEAN | M+NH4 MEAN | M+ Urea MEAN |
|---|---|---|---|---|---|---|
| 0 | 0.0 | 0.0 | 0.0 | 0.0 | 0.0 | 0.0 |
| 21 | 0.0 | 0.0 | 0.0 | 0.0 | 0.0 | 0.0 |
| 24 | 8.666666666666677 | 0.0 | 6.666666666666667 | 6.666666666666667 | 2.6666666666666665 | 1.3333333333333333 |
| 27 | 24.0 | 6.0 | 23.0 | 23.0 | 7.666666666666667 | 4.333333333333348 |
| 30 | 59.66666666666647 | 32.333333333333336 | 47.0 | 47.0 | 23.33333333333324 | 24.666666666666668 |
| 33 | 73.66666666666667 | 54.0 | 64.0 | 64.0 | 41.333333333333336 | 38.333333333333336 |
| 36 | 78.66666666666667 | 65.66666666666667 | 76.33333333333326 | 76.33333333333326 | 60.66666666666647 | 54.333333333333336 |
| 39 | 83.33333333333326 | 70.0 | 82.0 | 82.0 | 67.33333333333326 | 61.333333333333336 |
| 42 | 84.33333333333326 | 76.33333333333326 | 84.66666666666667 | 84.66666666666667 | 75.66666666666667 | 70.33333333333326 |
| 45 | 86.0 | 79.66666666666667 | 87.0 | 87.0 | 81.0 | 72.0 |
| 48 | 87.33333333333326 | 81.0 | 88.33333333333326 | 88.33333333333326 | 82.66666666666667 | 76.66666666666667 |
| 51 | 89.0 | 83.0 | 90.0 | 90.0 | 84.33333333333326 | 82.0 |
| 54 | 89.66666666666667 | 85.0 | 92.0 | 92.0 | 86.66666666666667 | 85.0 |
| 57 | 90.0 | 86.33333333333326 | 92.0 | 92.0 | 86.66666666666667 | 86.0 |
| 60 | 90.0 | 86.66666666666667 | 93.0 | 93.0 | 86.66666666666667 | 86.66666666666667 |
| 63 | 90.0 | 87.33333333333326 | 93.33333333333326 | 93.33333333333326 | 87.33333333333326 | 88.33333333333326 |
| 66 | 91.0 | 88.33333333333326 | 94.66666666666667 | 94.66666666666667 | 89.33333333333326 | 90.66666666666667 |
| 69 | 94.33333333333326 | 90.0 | 95.66666666666667 | 95.66666666666667 | 91.33333333333326 | 93.33333333333326 |
| 72 | 95.66666666666667 | 90.0 | 96.66666666666667 | 96.66666666666667 | 92.66666666666667 | 96.0 |
### Chart: Krishnahamsa
| Category | DW MEAN | M+N mean | M-N MEAN | M+NH4 MEAN | M+NH4NO3 MEAN | M+ Urea MEAN |
|---|---|---|---|---|---|---|
| 0 | 0.0 | 0.0 | 0.0 | 0.0 | 0.0 | 0.0 |
| 21 | 0.0 | 0.0 | 0.0 | 0.0 | 0.0 | 0.0 |
| 24 | 0.0 | 0.0 | 0.0 | 0.0 | 0.0 | 0.0 |
| 27 | 0.0 | 0.0 | 0.0 | 0.0 | 0.0 | 0.0 |
| 30 | 0.0 | 0.0 | 0.0 | 0.3333333333333333 | 0.0 | 0.0 |
| 33 | 0.3333333333333333 | 0.0 | 0.0 | 0.6666666666666666 | 0.0 | 0.6666666666666666 |
| 36 | 7.666666666666667 | 1.0 | 5.333333333333348 | 5.0 | 2.3333333333333335 | 1.0 |
| 39 | 18.666666666666668 | 3.0 | 18.33333333333324 | 10.333333333333334 | 4.333333333333348 | 1.6666666666666667 |
| 42 | 37.66666666666647 | 11.666666666666694 | 35.0 | 17.0 | 12.333333333333334 | 3.6666666666666665 |
| 45 | 42.0 | 22.666666666666668 | 49.66666666666647 | 24.666666666666668 | 31.0 | 7.333333333333348 |
| 48 | 62.0 | 50.333333333333336 | 58.0 | 56.333333333333336 | 43.66666666666647 | 14.333333333333334 |
| 51 | 72.66666666666667 | 55.66666666666647 | 68.66666666666667 | 66.66666666666667 | 53.0 | 25.33333333333324 |
| 54 | 83.33333333333326 | 69.33333333333326 | 78.66666666666667 | 79.33333333333326 | 69.33333333333326 | 40.66666666666647 |
| 57 | 87.33333333333326 | 78.0 | 83.33333333333326 | 84.33333333333326 | 73.66666666666667 | 55.0 |
| 60 | 89.33333333333326 | 81.66666666666667 | 86.66666666666667 | 87.33333333333326 | 75.66666666666667 | 62.66666666666647 |
| 63 | 91.0 | 85.33333333333326 | 89.33333333333326 | 90.33333333333326 | 78.66666666666667 | 74.0 |
| 66 | 92.66666666666667 | 88.0 | 90.66666666666667 | 93.0 | 80.66666666666667 | 78.33333333333326 |
| 69 | 94.66666666666667 | 91.66666666666667 | 93.33333333333326 | 95.0 | 86.66666666666667 | 91.0 |
| 72 | 96.33333333333326 | 96.66666666666667 | 96.66666666666667 | 97.0 | 95.33333333333326 | 93.66666666666667 |
### Chart: Prasanna
| Category | DW MEAN | M+N mean | M-N MEAN | M+NH4 MEAN | M+NH4NO3 MEAN | M+ Urea MEAN |
|---|---|---|---|---|---|---|
| 0 | 0.0 | 0.0 | 0.0 | 0.0 | 0.0 | 0.0 |
| 21 | 0.0 | 0.0 | 0.0 | 0.0 | 0.0 | 0.3333333333333333 |
| 24 | 3.3333333333333335 | 0.3333333333333333 | 2.0 | 1.3333333333333333 | 0.6666666666666666 | 1.0 |
| 27 | 13.0 | 5.666666666666667 | 8.0 | 12.333333333333334 | 5.0 | 4.333333333333348 |
| 30 | 50.66666666666647 | 15.666666666666694 | 36.0 | 35.0 | 23.666666666666668 | 12.666666666666694 |
| 33 | 79.33333333333326 | 43.333333333333336 | 77.33333333333326 | 69.0 | 59.333333333333336 | 29.33333333333324 |
| 36 | 92.0 | 74.33333333333326 | 91.33333333333326 | 83.66666666666667 | 85.33333333333326 | 65.0 |
| 39 | 95.0 | 89.33333333333326 | 97.0 | 93.0 | 91.33333333333326 | 81.33333333333326 |
| 42 | 97.33333333333326 | 94.0 | 98.66666666666667 | 95.33333333333326 | 95.66666666666667 | 92.66666666666667 |
| 45 | 97.33333333333326 | 97.66666666666667 | 100.0 | 99.33333333333326 | 98.66666666666667 | 96.33333333333326 |
| 48 | 97.33333333333326 | 98.66666666666667 | 100.0 | 99.33333333333326 | 98.66666666666667 | 100.0 |
| 51 | 97.33333333333326 | 98.66666666666667 | 100.0 | 99.33333333333326 | 98.66666666666667 | 100.0 |
| 54 | 97.33333333333326 | 98.66666666666667 | 100.0 | 99.33333333333326 | 98.66666666666667 | 100.0 |
| 57 | 97.33333333333326 | 98.66666666666667 | 100.0 | 99.33333333333326 | 98.66666666666667 | 100.0 |
| 60 | 97.33333333333326 | 98.66666666666667 | 100.0 | 99.33333333333326 | 98.66666666666667 | 100.0 |
| 63 | 97.33333333333326 | 98.66666666666667 | 100.0 | 99.33333333333326 | 98.66666666666667 | 100.0 |
| 66 | 97.33333333333326 | 98.66666666666667 | 100.0 | 99.33333333333326 | 98.66666666666667 | 100.0 |
| 69 | 97.33333333333326 | 98.66666666666667 | 100.0 | 99.33333333333326 | 98.66666666666667 | 100.0 |
| 72 | 97.33333333333326 | 98.66666666666667 | 100.0 | 99.33333333333326 | 98.66666666666667 | 100.0 |

## Slide 7
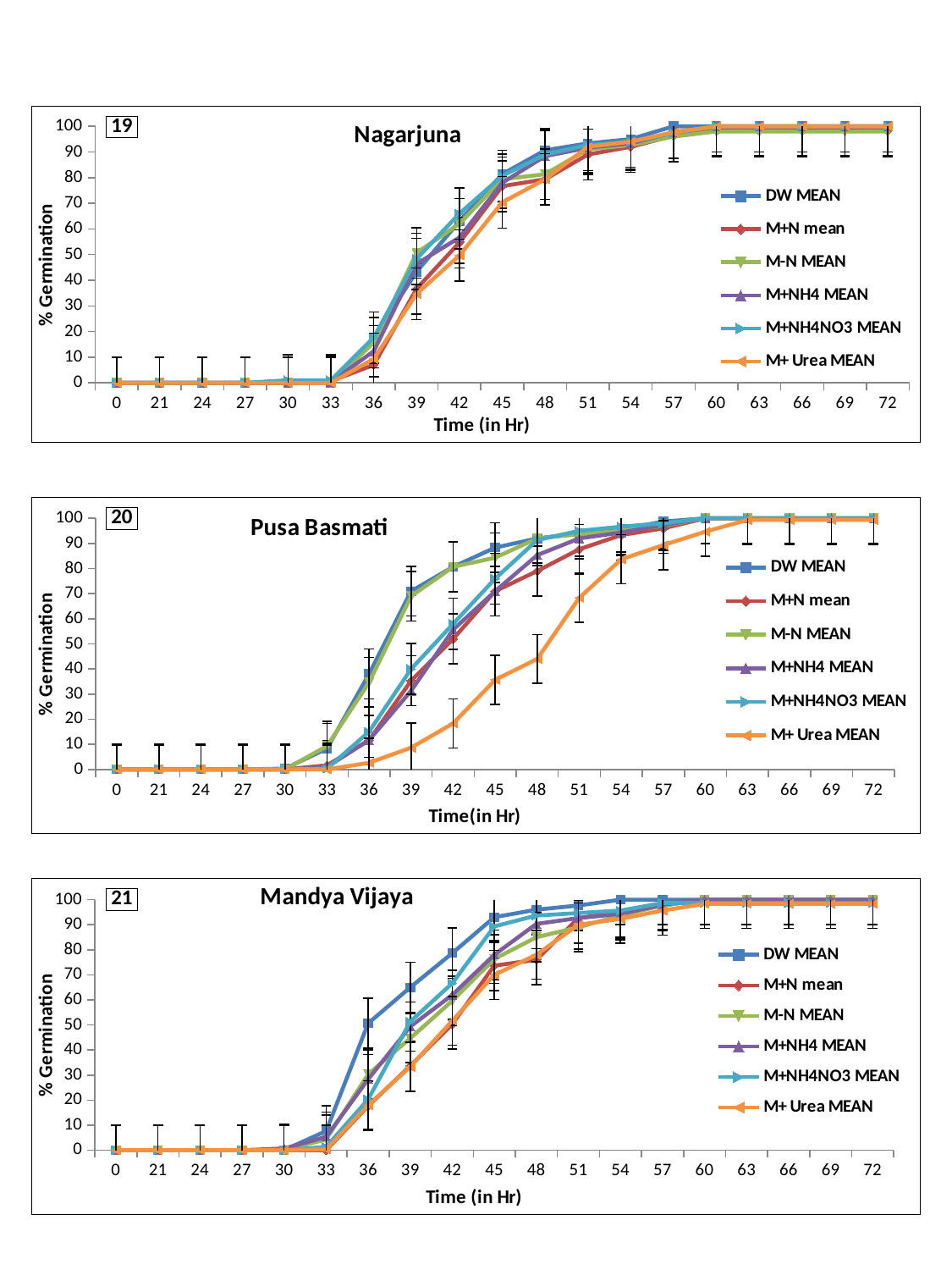

### Chart: Nagarjuna
| Category | DW MEAN | M+N mean | M-N MEAN | M+NH4 MEAN | M+NH4NO3 MEAN | M+ Urea MEAN |
|---|---|---|---|---|---|---|
| 0 | 0.0 | 0.0 | 0.0 | 0.0 | 0.0 | 0.0 |
| 21 | 0.0 | 0.0 | 0.0 | 0.0 | 0.0 | 0.0 |
| 24 | 0.0 | 0.0 | 0.0 | 0.0 | 0.0 | 0.0 |
| 27 | 0.0 | 0.0 | 0.0 | 0.0 | 0.0 | 0.0 |
| 30 | 0.0 | 0.0 | 0.0 | 0.0 | 1.0 | 0.0 |
| 33 | 0.0 | 0.6666666666666666 | 0.0 | 0.3333333333333333 | 1.0 | 0.0 |
| 36 | 17.666666666666668 | 7.0 | 15.666666666666694 | 12.333333333333334 | 17.666666666666668 | 9.333333333333336 |
| 39 | 43.333333333333336 | 36.66666666666647 | 50.66666666666647 | 46.333333333333336 | 48.333333333333336 | 34.66666666666647 |
| 42 | 63.0 | 54.66666666666647 | 62.0 | 56.66666666666647 | 66.0 | 49.66666666666647 |
| 45 | 81.33333333333326 | 76.66666666666667 | 79.33333333333326 | 78.0 | 80.66666666666667 | 70.33333333333326 |
| 48 | 90.66666666666667 | 79.33333333333326 | 81.33333333333326 | 88.33333333333326 | 89.0 | 79.33333333333326 |
| 51 | 93.33333333333326 | 89.0 | 91.0 | 91.66666666666667 | 92.66666666666667 | 92.0 |
| 54 | 95.0 | 92.0 | 92.66666666666667 | 93.33333333333326 | 94.0 | 94.0 |
| 57 | 100.0 | 96.33333333333326 | 96.0 | 97.66666666666667 | 97.33333333333326 | 97.66666666666667 |
| 60 | 100.0 | 98.33333333333326 | 98.0 | 100.0 | 100.0 | 100.0 |
| 63 | 100.0 | 98.33333333333326 | 98.0 | 100.0 | 100.0 | 100.0 |
| 66 | 100.0 | 98.33333333333326 | 98.0 | 100.0 | 100.0 | 100.0 |
| 69 | 100.0 | 98.33333333333326 | 98.0 | 100.0 | 100.0 | 100.0 |
| 72 | 100.0 | 98.33333333333326 | 98.0 | 100.0 | 100.0 | 100.0 |
### Chart: Pusa Basmati
| Category | DW MEAN | M+N mean | M-N MEAN | M+NH4 MEAN | M+NH4NO3 MEAN | M+ Urea MEAN |
|---|---|---|---|---|---|---|
| 0 | 0.0 | 0.0 | 0.0 | 0.0 | 0.0 | 0.0 |
| 21 | 0.0 | 0.0 | 0.0 | 0.0 | 0.0 | 0.0 |
| 24 | 0.0 | 0.0 | 0.0 | 0.0 | 0.0 | 0.0 |
| 27 | 0.0 | 0.0 | 0.0 | 0.0 | 0.0 | 0.0 |
| 30 | 0.3333333333333333 | 0.0 | 0.0 | 0.0 | 0.0 | 0.0 |
| 33 | 8.333333333333336 | 1.6666666666666667 | 9.333333333333336 | 1.0 | 0.3333333333333333 | 0.0 |
| 36 | 38.0 | 11.666666666666694 | 34.66666666666647 | 11.666666666666694 | 15.0 | 2.6666666666666665 |
| 39 | 71.0 | 35.333333333333336 | 69.0 | 31.33333333333324 | 40.0 | 8.666666666666677 |
| 42 | 80.66666666666667 | 52.0 | 80.66666666666667 | 55.66666666666647 | 58.0 | 18.33333333333324 |
| 45 | 88.33333333333326 | 71.0 | 84.33333333333326 | 71.0 | 76.0 | 35.66666666666647 |
| 48 | 92.0 | 79.0 | 92.0 | 85.33333333333326 | 91.33333333333326 | 44.0 |
| 51 | 94.0 | 87.66666666666667 | 93.66666666666667 | 92.0 | 95.0 | 68.33333333333326 |
| 54 | 95.66666666666667 | 93.33333333333326 | 95.0 | 94.33333333333326 | 96.66666666666667 | 83.66666666666667 |
| 57 | 98.66666666666667 | 96.0 | 97.0 | 97.33333333333326 | 98.0 | 89.33333333333326 |
| 60 | 100.0 | 100.0 | 100.0 | 100.0 | 100.0 | 94.66666666666667 |
| 63 | 100.0 | 100.0 | 100.0 | 100.0 | 100.0 | 99.33333333333326 |
| 66 | 100.0 | 100.0 | 100.0 | 100.0 | 100.0 | 99.33333333333326 |
| 69 | 100.0 | 100.0 | 100.0 | 100.0 | 100.0 | 99.33333333333326 |
| 72 | 100.0 | 100.0 | 100.0 | 100.0 | 100.0 | 99.33333333333326 |
### Chart: Mandya Vijaya
| Category | DW MEAN | M+N mean | M-N MEAN | M+NH4 MEAN | M+NH4NO3 MEAN | M+ Urea MEAN |
|---|---|---|---|---|---|---|
| 0 | 0.0 | 0.0 | 0.0 | 0.0 | 0.0 | 0.0 |
| 21 | 0.0 | 0.0 | 0.0 | 0.0 | 0.0 | 0.0 |
| 24 | 0.0 | 0.0 | 0.0 | 0.0 | 0.0 | 0.0 |
| 27 | 0.0 | 0.0 | 0.0 | 0.0 | 0.0 | 0.0 |
| 30 | 0.0 | 0.0 | 0.0 | 0.6666666666666666 | 0.0 | 0.0 |
| 33 | 7.666666666666667 | 0.0 | 4.333333333333348 | 5.333333333333348 | 1.3333333333333333 | 0.3333333333333333 |
| 36 | 50.66666666666647 | 17.666666666666668 | 30.33333333333324 | 28.33333333333324 | 20.33333333333324 | 18.0 |
| 39 | 65.0 | 33.66666666666647 | 44.66666666666647 | 49.333333333333336 | 51.333333333333336 | 33.333333333333336 |
| 42 | 78.66666666666667 | 50.333333333333336 | 59.66666666666647 | 62.0 | 66.66666666666667 | 51.66666666666647 |
| 45 | 93.0 | 73.66666666666667 | 76.33333333333326 | 78.0 | 89.33333333333326 | 70.0 |
| 48 | 96.0 | 76.0 | 85.0 | 90.33333333333326 | 93.66666666666667 | 78.0 |
| 51 | 97.66666666666667 | 92.66666666666667 | 89.0 | 92.66666666666667 | 94.66666666666667 | 90.0 |
| 54 | 100.0 | 95.0 | 93.66666666666667 | 94.33333333333326 | 95.66666666666667 | 92.33333333333326 |
| 57 | 100.0 | 97.66666666666667 | 97.66666666666667 | 98.0 | 98.66666666666667 | 95.66666666666667 |
| 60 | 100.0 | 100.0 | 100.0 | 100.0 | 98.66666666666667 | 98.33333333333326 |
| 63 | 100.0 | 100.0 | 100.0 | 100.0 | 98.66666666666667 | 98.33333333333326 |
| 66 | 100.0 | 100.0 | 100.0 | 100.0 | 98.66666666666667 | 98.33333333333326 |
| 69 | 100.0 | 100.0 | 100.0 | 100.0 | 98.66666666666667 | 98.33333333333326 |
| 72 | 100.0 | 100.0 | 100.0 | 100.0 | 98.66666666666667 | 98.33333333333326 |
