# Supplementary material for: Phenotyping for Nitrogen Use Efficiency: Rice Genotypes Differ in N-Responsive Germination, Oxygen Consumption, Seed Urease Activities, Root Growth, Crop Duration, and Yield at Low N
Source: Front Plant Sci. 2018 Oct 1;9:1452. doi: 10.3389/fpls.2018.01452 (PMC6174359; doi:10.3389/fpls.2018.01452)
Supplement: TABLE S1 — Correlation analysis ofthe ranking of 21 geno types by Kendall’s tau_b test. (a) Ranking based on time taken for 50% seeds to germinate (X0) and (b) ranking based on the effect of N on the time taken for 50% seeds to germinate (ΔX0). X0 values were obtained as described in the legend and the difference between the X0 values with and without N were calculated as ΔX0. Values in the parenthesis shows significance. [file Table_1.docx]

**Supplementary Table 1a:**

| Kendall’s tau_b Correlations on X_0_ ranking: Significant at the 0.01 level (2-tailed) | | | | | | | |
| --- | --- | --- | --- | --- | --- | --- | --- |
| Kendall’s tau_b | Treatments |  | M-Nitrogen | M+NH_4_Cl | M+NH_4_NO_3_ | M+NO_3_ | M+Urea |
|  | M-Nitrogen | Correlation coefficient | 1.000 | .781  (<0.001) | .874  (<0.001) | .876  (<0.001) | .829  (<0.001) |
|  |  | N | 21 | 21 | 21 | 21 | 21 |
|  | M+NH_4_Cl | Correlation coefficient | .781  (<0.001) | 1.000 | .807  (<0.001) | .790  (<0.001) | .838  (<0.001) |
|  |  | N | 21 | 21 | 21 | 21 | 21 |
|  | M+NH_4_NO_3_ | Correlation coefficient | .874  (<0.001) | .807  (<0.001) | 1.000 | .893  (<0.001) | .833  (<0.001) |
|  |  | N | 21 | 21 | 21 | 21 | 21 |
|  | M+NO_3_ | Correlation coefficient | .876  (<0.001) | .790  (<0.001) | .893  (<0.001) | 1.000 | .883  (<0.001) |
|  |  | N | 21 | 21 | 21 | 21 | .000 |
|  | M+Urea | Correlation coefficient | .829  (<0.001) | .838  (<0.001) | .883  (<0.001) | .838  (<0.001) | 21 |
|  |  | N | 21 | 21 | 21 | 21 | 21 |

**Supplementary Table.1b:**

| Kendall’s tau_b Correlations on ∆X_0_ ranking: Significant at the 0.01 level (2-tailed). | | | | | | |
| --- | --- | --- | --- | --- | --- | --- |
| Kendall’s tau_b | Treatments |  | ∆M+NO_3_ | ∆M+NH_4_Cl | ∆M+Urea | ∆M+NH_4_NO_3_ |
|  | ∆M+NO_3_ | Correlation coefficient | 1.000 | -0.48  (ns) | 0.495  (<0.01) | .571  (<0.001) |
|  |  | N | 21 | 21 | 21 | 21 |
|  | ∆M+NH_4_Cl | Correlation coefficient | -0.48  (ns) | 1.000 | -.171  (ns) | -.190  (ns) |
|  |  | N | 21 | 21 | 21 | 21 |
|  | ∆M+Urea | Correlation coefficient | 0.495  ((<0.01) | -.171  (ns) | 1.000 | .676  (<0.001) |
|  |  | Sig.(two tailed) | .002 | .277 |  | .000 |
|  |  | N | 21 | 21 | 21 | 21 |
|  | ∆M+NH_4_NO_3_ | Correlation coefficient | .571  (<0.001) | -.190  (ns) | .676  (<0.001) | 1.000 |
|  |  | N | 21 | 21 | 21 | 21 |

**Supplementary Table:1. Correlation analysis of the ranking of 21 genotypes** **by** **Kendall’s tau_b test. a.** Ranking based on time taken for 50% seeds to germinate (X0) and **b.** Ranking based on the effect of N on the time taken for 50% seeds to germinate (∆X0)**.** X0 values were obtained as described in the legend for supplementary Table 1 and the difference between the X0 values with and without N were calculated as ∆X0. Values in the parenthesis shows significance.
