# Supplementary material for: Phenotyping for Nitrogen Use Efficiency: Rice Genotypes Differ in N-Responsive Germination, Oxygen Consumption, Seed Urease Activities, Root Growth, Crop Duration, and Yield at Low N
Source: Front Plant Sci. 2018 Oct 1;9:1452. doi: 10.3389/fpls.2018.01452 (PMC6174359; doi:10.3389/fpls.2018.01452)
Supplement: TABLE S2 — Mean field data for grain yield and crop duration for 15 rice geno types grown without added N(N0)or with 100 Kg/ha urea N(N100) for 3 years over 6 seasons (2 seasons/year, Rabi and Kharif). [file Table_2.docx]

Supplementary Table 2:

|  | **Grain yield (t/ha)** | | **Crop duration (Days)** |
| --- | --- | --- | --- |
| **Genotypes** | **N-0 (no nitrogen)** | **N100 (N-100 kg/ha)** |  |
| Aditya | 2.93 | 4.44 | 120 |
| Swarnadhan | 3.63 | 5.55 | 120 |
| Rasi | 3.13 | 4.85 | 120 |
| Jaya | 3.86 | 6.03 | 130 |
| Varadhan | 3.80 | 6.13 | 130 |
| Ravi | 3.08 | 4.64 | 120 |
| Swarna | 4.07 | 5.06 | 150 |
| Suraksha | 3.08 | 4.94 | 135 |
| Vibhava | 3.35 | 5.40 | 125 |
| Vikas | 3.45 | 5.48 | 115 |
| Krishna Hamsa | 3.52 | 4.81 | 125 |
| Sampada | 4.02 | 5.50 | 140 |
| Prasanna | 2.91 | 3.73 | 105 |
| Pusa Basmati | 3.65 | 4.86 | 130 |
| Triguna | 3.56 | 4.53 | 125 |

**Supplementary Table 2. Mean field data for** grain yield and crop duration for 15 rice genotypes grown without added N (N0) or with 100 Kg/ha urea N (N100) for 3 years over 6 seasons (2 seasons/year, Rabi and Kharif).
